# Combining SPR with atomic-force microscopy enables single-molecule insights into activation and suppression of the complement cascade

**DOI:** 10.1074/jbc.RA119.010913

**Published:** 2019-11-12

**Authors:** Elisavet Makou, Richard G. Bailey, Heather Johnston, John D. Parkin, Alison N. Hulme, Georg Hähner, Paul N. Barlow

**Affiliations:** ‡ EaStChem School of Chemistry, University of Edinburgh, Joseph Black Chemistry Building, Edinburgh, Scotland EH9 3FJ, United Kingdom; § EaStChem School of Chemistry, University of St. Andrews, St Andrews, Scotland KY16 9ST, United Kingdom; ¶ School of Biological Sciences, University of Edinburgh, Edinburgh, Scotland EH9 3JY, United Kingdom

**Keywords:** surface plasmon resonance (SPR), atomic force microscopy (AFM), complement system, protein-protein interaction, protein conformation, inflammation, single-molecule biophysics, C3b, factor H, immune response, molecular stretching, self-assembling monolayer, single-molecule analysis

## Abstract

Activation and suppression of the complement system compete on every serum-exposed surface, host or foreign. Potentially harmful outcomes of this competition depend on surface molecules through mechanisms that remain incompletely understood. Combining surface plasmon resonance (SPR) with atomic force microscopy (AFM), here we studied two complement system proteins at the single-molecule level: C3b, the proteolytically activated form of C3, and factor H (FH), the surface-sensing C3b-binding complement regulator. We used SPR to monitor complement initiation occurring through a positive-feedback loop wherein surface-deposited C3b participates in convertases that cleave C3, thereby depositing more C3b. Over multiple cycles of flowing factor B, factor D, and C3 over the SPR chip, we amplified C3b from ∼20 to ∼220 molecules·μm^−2^. AFM revealed C3b clusters of up to 20 molecules and solitary C3b molecules deposited up to 200 nm away from the clusters. A force of 0.17 ± 0.02 nanonewtons was needed to pull a single FH molecule, anchored to the AFM probe, from its complex with surface-attached C3b. The extent to which FH molecules stretched before detachment varied widely among complexes. Performing force-distance measurements with FH(D1119G), a variant lacking one of the C3b-binding sites and causing atypical hemolytic uremic syndrome, we found that it detached more uniformly and easily. In further SPR experiments, *K_D_* values between FH and C3b on a custom-made chip surface were 5-fold tighter than on commercial chips and similar to those on erythrocytes. These results suggest that the chemistry at the surface on which FH acts drives conformational adjustments that are functionally critical.

## Introduction

Within minutes of entering the human bloodstream, foreign material becomes coated with millions of copies of the protein C3b (1). C3b (177 kDa) is cleaved from the soluble plasma protein C3 (185 kDa), the most abundant among the 40 proteins of the complement system. The presence of numerous C3b molecules on a cell or particle tags it for clearance (2). At higher densities, C3b triggers a proteolytic cascade leading eventually, via cleavage of C5 to C5b, to formation of the potentially cytolytic membrane-attack complex (3). Cleavages of C3 and C5 also generate the pro-inflammatory anaphylatoxins, C3a and C5a (4).

**Figure 1.**
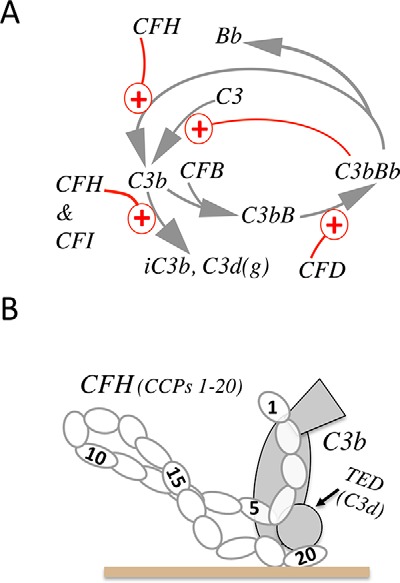
**Amplification loop and regulatory role of FH.** *A*, FH intercedes in the positive-feedback loop of the AP of complement activation by competing with FB for binding to C3b, accelerating decay of the convertase C3bBb, and recruiting factor I (FI) to C3b, whereupon FI cleaves C3b to iC3b (subsequently further cleaved to C3dg and C3d). *B*, FH consists of 20 CCP modules (*numbered*) and binds C3b (in fluid phase or on a surface) primarily via two sites, in CCPs 1–4 and CCPs 19–20. The C-terminal site binds to the TED of C3b that corresponds to C3d, the ultimate proteolytic degradation product of C3b.

The swiftness of this evolutionarily ancient response to invasion is vital and relies on the “C3b-amplification loop” (5) (Fig. 1*A*) that is core to all three activation pathways of the complement system (6, 7). Many human diseases are linked to inadequate regulation of C3b amplification on host surfaces (8). Numerous approaches are being explored to restore its regulation therapeutically (9), with some candidates well-advanced in clinical trials (10).

The C3b-amplification loop is initiated by small quantities of “seed” C3b that arise from continuous low-level fluid-phase proteolysis of C3 (see below). This ubiquitous spontaneous process underlies the “alternative” pathway (AP)^4^ of complement activation (11). Each nascent C3b molecule possesses a thioester group (12) that is exposed and activated during conversion of C3 to C3b (13). In its activated form, this thioester will react with hydroxyl groups in a predominantly indiscriminate fashion on virtually any surface, forming a covalent bond. Alternatively, the thioester gets hydrolyzed, producing soluble C3b(H_2_O) (14). Crucially C3b, on surfaces or in fluid phase, binds factor B (FB), which becomes proteolytically activated by factor D (FD) to yield C3bBb (Fig. 1*A*). This complex is called a C3 convertase because its Bb component cleaves C3 and hence generates additional C3b that can form more C3bBb. A related process generates the seed C3b mentioned above (15); very slow hydrolysis of the buried thioester within C3 forms C3(H_2_O) that structurally resembles and behaves like C3b (16) in that it can act as a platform for FB cleavage by FD forming C3(H_2_O)Bb in the fluid phase, which converts C3 to C3b and hence seeds the amplification loop.

Several crucial self-damping properties of the positive-feedback C3b-amplification loop ensure that complement activation remains localized to the initiation site. The C3b thioester bond is inherently labile, limiting how far nascent molecules diffuse before either binding to a surface or getting hydrolyzed (17, 18). Moreover, C3bBb (C3 convertase) turns over C3 relatively slowly (19), and the convertase irreversibly dissociates into C3b and Bb with a half-life of ∼90 s, although this is prolonged by the protein properdin (19, 20).

On most foreign surfaces, the outcome of the AP is normally rapid accumulation of covalently linked C3b. Thus, the speedy demise of invading organisms is assured, and the AP is a lag time–free, antibody-independent first line of defense. Conversely, on healthy host cells, the C3b-amplification loop is strongly suppressed. On damaged, diseased, or dying self cells and associated cellular debris and waste products (destined for clearance), C3b amplification is partially suppressed (21). Complement-regulating proteins are the key to this discrimination between self, nonself, and damaged self (22–25). For instance, factor H (FH) (26, 27), an abundant 155-kDa serum glycoprotein comprised of 20 similar compact modules, competes with FB for binding to C3b (Fig. 1*B*), thereby suppressing convertase formation. FH also accelerates the decay of C3bBb (Fig. 1*A*). Once bound to C3b, FH recruits factor I (FI) that cleaves C3b to C3f and iC3b. iC3b may be further sequentially degraded to C3dg and C3d that remain surface-bound. Crucially, FH has the key property of efficiently regulating C3b amplification on self surfaces but not on foreign ones (28). On self-surfaces, FH recognizes sialic acids and glycosaminoglycans as well as iC3b and C3d (*i.e.* remnants of a previous regulation event) (27, 29). Thus, FH selectively protects self-cells, although some bacteria can hijack FH (30). FH also promotes clearance of damaged self, but in a noninflammatory manner (21). Here it partly suppresses amplification such that less C3b, and hence less C3a and C5a, are produced, whereas the key proteolytic product is iC3b. iC3b is important for phagocytosis and binds to receptors with immunosuppressive properties (31). Other complement-regulating proteins, many with related structures and modes of action (22), are attached to or buried in host cell membranes.

Despite 50 years of effort, C3b amplification and the role of FH remain incompletely understood. Questions remain about the dynamic molecular landscape in which FH must operate to achieve control of the process. Whereas low numbers of seed C3b occur on every surface, clusters of C3b are believed to form during early-stage amplification, wherein C3b progeny surround parental C3 convertases. Although their existence has been inferred from observations of ferritin-conjugated anti-C3b antibodies (32), clusters have not been visualized directly. How quickly do they grow and spread? Do C3b molecules within a cluster bind each other, forming a mound, or spread out, forming a one-molecule-thick layer on the surface? Crucially, the mechanism whereby FH focuses its regulatory actions on self cells rather than foreign ones remains unclear. Early models invoked selective engagement of FH with C3b on a self-surface enhanced by co-binding of nearby markers, such as sialic acids, glycosaminoglycans, and C3b fragments (24). More recent models hypothesize critical conformational changes in FH (27, 33, 34).

Herein we establish the utility of combining SPR and AFM to gain insights into C3b amplification and its regulation. We observed and counted C3b molecules using AFM, following a C3b-amplification event controlled and monitored in the SPR instrument. AFM was also used to detect molecular stretching and measure forces involved in pulling FH molecules out of their complexes with C3b. By incorporating into the study custom-made SPR chips and disease-related and truncation FH mutants, we investigated the roles of surface chemistry, conformational flexibility, and the two C3b-binding sites of FH.

## Results

### AFM of C3b molecules after deposition on SPR sensor chip

We combined SPR and AFM to study the propagation on a surface of covalently attached C3b molecules (Fig. 1*A*). In SPR, responses are directly proportional to the accumulated mass of molecules on the sensor chip. SPR can thus be used to quantitatively monitor in real time the arrival on the surface of C3b molecules, generated *in situ*. If the surface bears hydroxyl groups to which nascent C3b can form a chemical bond, the response observed will represent the sum of C3b binding covalently, plus proteins associating reversibly with the surface. Thus, after washing, remaining signal will originate solely from covalently bound C3b. To count these molecules and assess their distribution, we utilized AFM. We used C1 sensor chips because they lack dextran and have a flat AFM-compatible surface.

**Figure 2.**
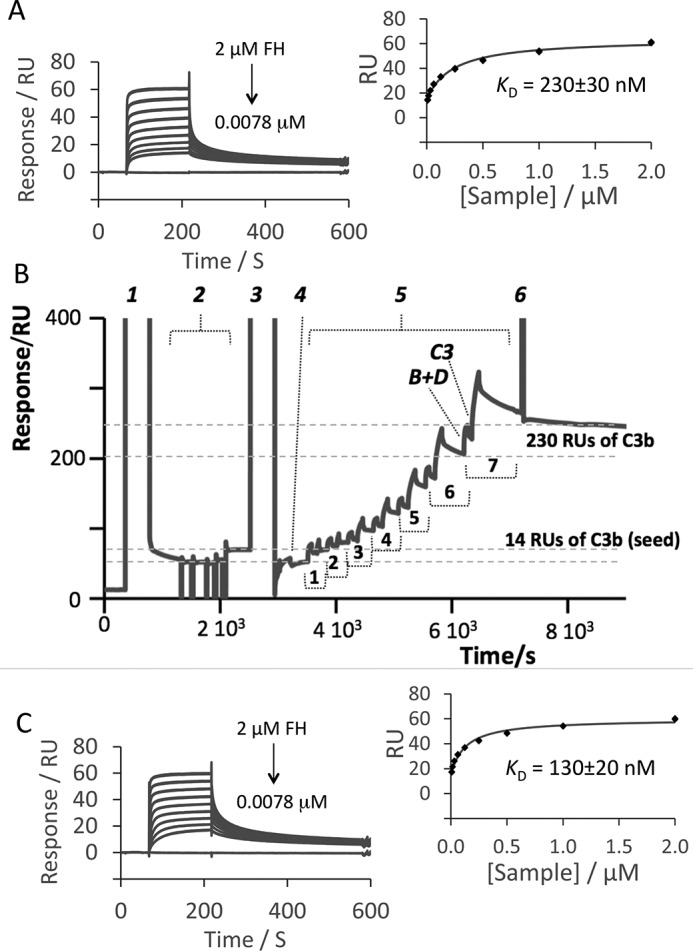
**SPR traces.** *A*, using amine coupling, 144 RU of C3b were immobilized on a C1 chip. The SPR responses measured while flowing a 2-fold dilution series of FH solutions over this surface are shown. The deposited C3b bound FH with the expected affinity (*inset*) (see Table 1). *B*, representative SPR trace for physiological immobilization of C3b on a C1 chip. Injections were as follows: *1*, EDC/NHS (for activation); *2*, C3b (amine coupling, several injections to achieve 14 RU); *3*, ethanolamine; *4*, buffer; *5*, seven cycles (numbered) of flowing 50 μm FB mixed with 50 μm FD (*B*+*D*) followed by 50 μm C3 (*3*), thus immobilizing ∼200 further RU of C3b; *6*, 3 m NaCl wash. *C*, SPR traces for a dilution series of FH solutions flowed over C3b immobilized on a C1 chip prepared as shown in *B*. The inferred *K_D_* value is listed in Table 1.

Initially, we aimed to image preformed C3b molecules after random amine–coupling them to the surface. We coupled 144 response units (RU) of C3b to flow channel 2 of a C1 chip in the SPR instrument, leaving channel 1 C3b-free. To assess the presence of functional C3b, we flowed over channel 2 a concentration series of FH solutions, subtracted the signals from channel 1, and inferred a *K_D_* assuming a 1:1 complex. This lay within the previously reported range (Fig. 2*A* and Table 1) (35–37). The extrapolated maximum response (*R*_max_), of ∼50 RU corresponds to saturation of FH-binding sites on C3b molecules in the channel. Assuming proportionality between mass and RUs, and based on molecular mass ratios (FH 155 kDa, C3b 177 kDa), *R*_max_ would be ∼125 RU were each C3b molecule to bind a FH molecule (155 kDa/177 kDa × 144 RU of deposited C3b). Conceivably, only ∼40% (*i.e.* 100 × 50/125) of amine-coupled C3b molecules are orientated in a way that enables them to interact with FH, or, given that FH has primary C3b-binding sites at either terminus (35) (Fig. 1*B*) and a potential third C3b-binding site in its seventh CCP module (38, 39), some FH molecules might bind two C3b molecules; formation of a mixture of 1:1 and 1:2 FH:C3b complexes could explain the imperfect fit to a 1:1 model seen in Fig. 2*A*.

**Table 1:** **Summary of the outcomes of SPR experiments on C1 and HJEM-series chips**

Chip type (figure)	Sample immobilized	Sample injected	*K_D_* ± S.E.*^a^*	Offset	χ^2^/*R*_max_
			μ*m*	*RU*	*(RU^2^)/RU*
C1 (Fig. 2*A*)	C3b (amine)*^b^*; 144 RU	FH (2 μm -7.8 nm)	0.23 ± 0.03*^c^*	14.9	2.8/49.2
C1 (Fig. 2*C*)	C3b (physiological); 196 RU	FH (2 μm to 7.8 nm)	0.13 ± 0.02*^c^*	14.7	5.7/45.1
HJEM2*^d^* (Fig. 6*A*)	C3b (amine); 313 RU	FH (2 μm to 3.9 nm)	0.05 ± 0.01*^c^*	38.4	10.9/75.0
HJEM2 (Fig. 6*B*)	C3d (amine); 61 RU	FH (2 μm to 3.9 nm)	0.64 ± 0.06*^c^*	2.1	1.4/61.2
HJEM4 (Fig. 6*E*)	C3b (thiol); 240 RU	FH (2 μm to 3.9 nm)	0.18 ± 0.02*^e^*	9.7	4.6/61.2
HJEM6 (Fig. 6*F*)	FH-PspCN; 18 RU PspCN; 54 RU FH	C3b (1 μm to 3.9 nm)	0.07 ± 0.01*^e^*	2.0	0.3/18.9
HJEM7	C3b (amine); 144 RU	FH (2 μm to 3.9 nm)	0.07 ± 0.01*^e^*	17.1	4.2/57.8
HJEM7 (Fig. 6*D*)	C3b (amine)-3′SL (click); 143 RU C3b; 174 RU SL	FH (2 μm to 3.9 nm)	0.09 ± 0.01*^e^*	18.4	6.7/58.4
HJEM7 (Fig. 6*C*)	C3b (amine); 144 RU	FH 19–20 (10 μm to 3.9 nm)	0.35 ± 0.04*^e^*	2.2	0.1/7.3
HJEM7	C3b (amine)-3′SL (click); 143 RU C3b; 174 RU SL	FH 19–20 (10 μm to 3.9 nm)	2.6 ± 0.6*^e^*	3.6	0.6/15.0

*^a^* S.E. values derived from fitting the data using Biacore software.

*^b^* Amine, standard amine coupling; SH, coupling via maleimide; 3'SL (click), 3' sialyllactose attached using click chemistry; physiological, see legend to Fig. 2*C*; RU, response unit.

*^c^* Representative data from two or more SPR experiments (in each experiment, duplicate injections at each concentration were performed).

*^d^* For specific HJEM chip composition, see Table 2.

*^e^* One SPR experiment, with duplicate injections at each concentration, was performed.

**Figure 3.**
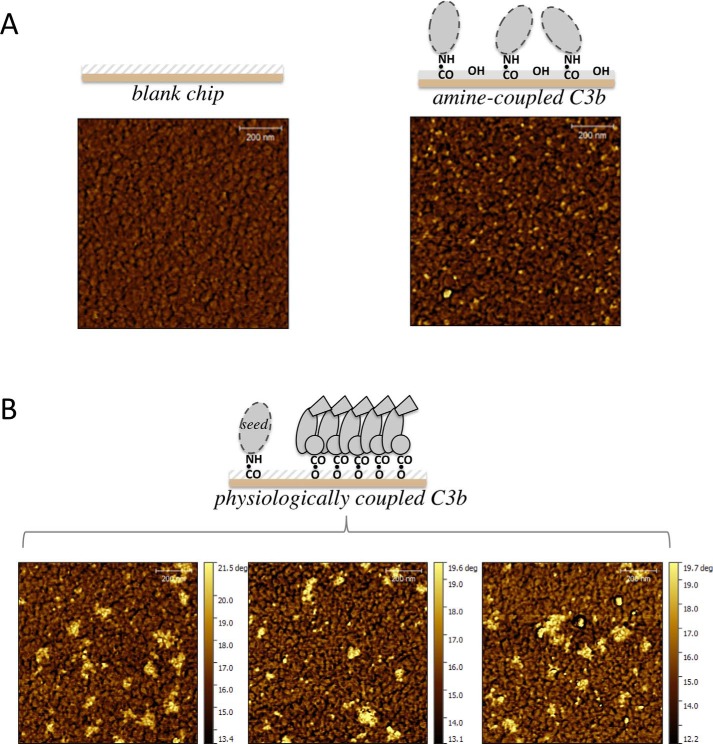
**Imaging C3b molecules on an SPR sensor chip.** *A*, a representative AFM phase image of a region of the C1 chip carrying 144 RU of amine-coupled C3b (*right*) compared with a no-C3b blank surface of the same chip (*left*). *B*, three examples of AFM phase images to show the complex C3b molecule landscape resulting from the multiple cycles of injections shown in Fig. 2*B*.

Another C1 chip was identically decorated with 144 RU of preformed C3b molecules within the SPR instrument but subsequently removed carefully from its cassette for AFM. Fig. 3*A* compares AFM images of C3b-free flow channel 1, and channel 2 that bears 144 RU of C3b. The C3b molecules appear on the flat surface of channel 2 as scattered objects, absent in channel 1. Another flow channel, decorated with 290 RU of C3b, appeared 2-fold more densely populated (Fig. S1). The diameters of these objects, 12.5 ± 0.3 nm (*n* = 80), are comparable with the dimensions of a C3b molecule (∼10 × 15 × 8 nm) (13). The low height of each object (<6 nm) was attributed to molecules drying out and collapsing onto the surface. Thus, most objects in these AFM images may be attributed to individual C3b molecules. There are examples of neighboring C3b molecules <60 nm apart and some objects that could be C3b dimers (highlighted in Fig. S1), consistent with the possibility of FH molecules being able to bind two C3b molecules on this surface.

To improve reliability of counting molecules, we adopted a procedure using phase images. “Soft” proteins showed up well against the background in phase images (Fig. S1). The summed surface area, within a 1 × 1-μm square of the AFM image, for all signals above a phase threshold (the minimum level that leaves only proteins visible) was assessed using Gwyddion software. This was divided by the mean surface area of individual C3b molecules (12.5-nm diameter circles), yielding 490 ± 20 C3b molecules·μm^−2^ for the 290-RU chip (mean ± S.D., *n* = 5 1 × 1-μm squares), in reasonable agreement with manual counting for an example square (Fig. S1). Because SPR correlates with number of surface molecules, a ratio of 1.7 (490/290) C3b molecules·μm^−2^ per RU was derived and used in further experiments. Our AFM-assisted C3b count indicates that a rule-of-thumb equation in which 1 RU = 1 pg·μm^−2^ (290 RU = 870 C3b molecules·μm^−2^) would overestimate the number of bound C3b molecules by ∼80%.

### C3b clusters formed in SPR experiments were imaged by AFM

We sought to deposit C3b on a C1 chip in a physiologically relevant way, using a modified published procedure (40) to generate the SPR traces in Fig. 2*B* (another example is shown in Fig. S2). We amine-coupled to the chip 14 RU of preformed C3b (Fig. 2*B*) as a “seed” and then injected a mixture of FB and FD. We expected FD to rapidly cleave FB within the surface-bound C3bB complex, yielding C3bBb, the C3 convertase. After eluting FD and Ba (and any intact FB), but before all surface-bound C3bBb dissociated into C3b (that stays covalently attached) and soluble Bb, we flowed C3 over this chip. In a recapitulation of the AP (Fig. 1*A*), surviving C3bBb on the surface should cleave this fresh supply of C3 to C3b. The nascent C3b will either hydrolyze and be eluted or will react via its activated thioester with the surface. Over further cycles of pulsing FB/FD and C3, injection durations were varied to adjust the final RU of accumulated C3b to ∼220, similar to the values achieved by amine coupling in the previous section.

For each cycle, the SPR trace (Fig. 2*B* and Fig. S2) reflects (i) a rise and fall in RU due to formation of surface-bound C3bBb (forms rapidly from C3bB) and then its partial “decay” (loss of Bb); (ii) a second gain in RU accompanying C3 injection, being a combination of reversible C3 binding to C3bBb whereupon C3 is cleaved to C3b, and covalent deposition of some of that C3b on the surface; and (iii) a post-C3-injection decline in RU explained by the continuing departure of Bb (from C3bBb) along with any free C3 and hydrolyzed C3b. A final high-salt wash removes noncovalently associated proteins, yielding a stable SPR signal arising from the original seed C3b plus a growing number of thioester-immobilized C3b molecules. These accumulated over seven cycles, corresponding to a net gain of ∼205 RU. Two C3b-decorated chips were identically prepared in this way. One was used to determine by SPR a *K_D_* for FH binding to predominantly physiologically immobilized C3b (Fig. 2*C*) on a C1 chip; the other was removed and imaged by AFM (Fig. 3*A*).

A *K_D_* = 130 nm for FH binding to C3b on this surface was estimated from SPR data (compared with *K_D_* = 230 nm (Table 1) for amine-coupled C3b). The extrapolated *R*_max_ was 50 RU, implying (as with amine-coupled C3b) either that a portion of C3b molecules are unavailable for binding or that one FH straddles two neighboring molecules. By AFM, physiologically deposited C3b molecules do not show the regular distribution seen after amine coupling. Instead, ∼80% lie in discrete, variably sized and shaped clusters. There are ∼25 clusters/μm^2^, with maximum dimensions of 50–200 nm. These are not tall enough to indicate C3b molecules stacked atop each another. Counting molecules within each cluster gave values of 3 to ∼20, with a median of 7 (Fig. S3). Remaining, isolated, C3b molecules are scattered, with some located 200 nm from a nearest neighbor.

The rate of C3b molecule deposition per convertase complex was approximated during the final cycle of the trace (Fig. 2*B*). Flowing FB and FD over the accumulated 165 RU of C3b (280 C3b molecule·μm^−2^, including 25 molecules of amine-coupled, seed C3b) yielded a 45-RU gain. This equates to up to ∼80% of C3b molecules binding FB, because if every 177-kDa C3b molecule had a bound 60-kDa Bb molecule, a gain of 60/177 kDa × 165 = 56 RU was expected (assuming rapid cleavage by FD in all cases). Hence, ∼225 (0.80 × 280) convertase complexes at most could have been formed·μm^−2^. In the 100-s delay before the C3 addition, about half of the convertases decayed, judging from the trace in Fig. 2*B* (as anticipated), leaving up to ∼110 intact convertases·μm^−2^. In the subsequent 60-s step of the cycle, convertases will have continued to dissociate but still generated 58 RU, or 100 molecules·μm^−2^, of immobilized C3b. It follows that between one and two C3b molecules were deposited s^−1^ per (surviving) C3bBb.

### Preparation of surfaces for adhesion-force measurements

We sought single-molecule information for the FH:C3b complex by attaching FH to the AFM probe tip. First we coated gold wafers and AFM cantilevers (note that the probe is part of the cantilever) by immersion in a solution of alkanethiols that adsorb to gold, forming a self-assembled monolayer (SAM). We used a mixture of alkanethiols (see Fig. S4) expected to give a SAM with 99 hydroxyl headgroups per carboxyl headgroup on its exposed surface. We immersed each SAM-coated wafer or cantilever in a sequence of solutions to achieve protein coupling. PspCN is a bacterial protein domain that binds to the middle region of FH tightly (34, 41). In most experiments, we initially thiol-coupled PspCN to maleimide headgroups of the SAM on the probe and then used the thiol-coupled PspCN as an anchor for FH. Alternatively, we amine-coupled FH directly to the SAM. We generally amine-coupled C3b to the SAM on the wafer.

Prior to attempting manipulations, we imaged these surfaces, after air drying, by AFM (Fig. S5). SAMs are invisible to contact-mode AFM; hence, the underlying structure of the gold surface is evident in SAM-only wafers, with striations corresponding to layering of the lattices within each gold grain. On C3b-coated wafers, a loss of visible topographical features of the gold indicated a dense layer of protein molecules (Fig. S5*A*). We could not image the CFH-decorated AFM probe tip itself, so we imaged the cantilever surface. On the cantilever surface bearing PspCN-anchored FH, AFM revealed numerous objects, many with a looplike appearance (Fig. S5, *B* and *C*). FH is an extended molecule that comprises 20 “CCP” (42) domains, each ∼4 nm long (43), and it has a compact, hinge-like central region (44, 45), so the looped features could be FH molecules. Their height of only a few nm suggests a partial collapse of the disulfide-stabilized protein on the air-dried chip. These observed high densities of C3b and FH molecules, on the wafer and probe, respectively, maximized the likelihood of productive intermolecular encounters during adhesion-force measurements.

### Force-distance curves for pulling a CFH molecule out of a C3b:CFH complex

**Figure 4.**
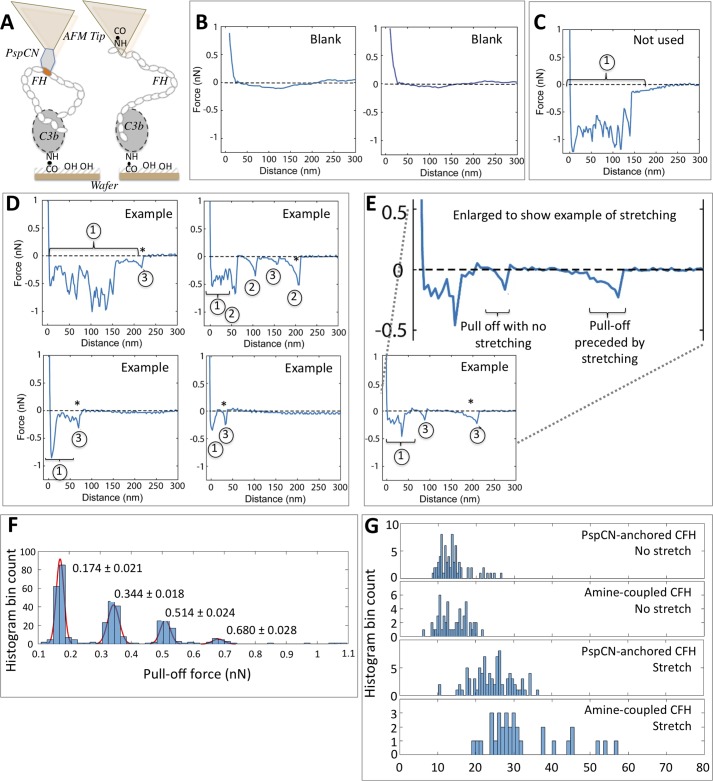
**Force-distance curves for disengaging FH from C3b.** *A*, FH was anchored to PspCN domains attached to a SAM formed on the AFM tip (*left*), or FH was randomly amine-coupled directly to a SAM on the AFM tip (*right*). C3b was amine-coupled to the SAM on the wafer. *B*, no-FH controls; the tip lacked PspCN. *C*, one of many force-distance curves collected using the setup in Fig. 4*A* (*left*). Because, in this example, the trace does not return abruptly to baseline, it was not analyzed further. *D*, additional examples of curves collected using the setup from *A* (*left*). Each trace corresponds to the separation of multiple FH and C3b molecules. Of the components within each curve, those labeled *1* were not interpreted. Those labeled *2* and *3*, after each of which the trace returns to the baseline, were considered further. Events labeled *3* could correspond to disengagement of one complex, whereas those labeled *2* correspond to two or more complexes. *E*, in this example, an event of type 3 involves molecular stretching prior to release. *F*, the forces for numerous events of types 2 and 3 are integral multiples of 0.17 nN. *G*, the distances over which many release events occurred were measured for both setups shown in Fig. 4*A*. Release events involving stretching (see *E*) were plotted separately. These are rarer and occur over longer distances for amine-coupled FH compared with PspCN-anchored FH.

To confirm reproducibility of adhesion-force measurements, we decorated multiple AFM probes with PspCN-anchored FH and used them to analyze the same C3b-coated wafer surface (Fig. 4*A*). Pull-off or detachment events were evident in >90% of the force-distance curves recorded as the FH-decorated probe was moved across the C3b-bearing surface. Reassuringly, we found that the adhesion parameters of individual detachment events (see below) remained consistent between identically prepared probes. We performed endurance tests to ascertain whether, over multiple measurements, FH is shed from the probe or C3b detached from the wafer surface. The same probe was used repeatedly, and pull-off forces over several hundred cycles were measured. No detectable deterioration occurred.

Fig. 4 shows examples of force-distance curves obtained using AFM probe tips decorated with PspCN-anchored FH or a tip devoid of PspCN that had been immersed in a FH solution (“blank”) (Fig. 4*B*). Further negative controls arose from decorating AFM probes with SAM only or with SAM plus PspCN (*i.e.* no FH). We detected no adhesion events for any of these. Relatively high surface densities of protein molecules on both tip and wafer account for why virtually all force-distance curves (Fig. 4, *C–E*), apart from blanks, are complex, corresponding to interactions between several molecules of FH on the tip and several of C3b on the wafer. We categorized the primary features of these force-distance curves into three types of event. An initial event (type 1), observed in most curves, has multiple components and is presumed to arise from numerous interactions; it was not further analyzed. Some subsequent detachment events, defined as types 2 and 3, end with complete relaxation (return to zero force) of the cantilever. These could correspond to individual (type 3) or multiple (type 2) FH molecules being detached from the C3b-decorated surface, whereas the final event in each curve (marked with an *asterisk* in Fig. 4) represents the very last FH molecule(s) becoming detached. We analyzed the forces associated with events of types 2 and 3 (*i.e.* only considering events where the cantilever relaxed to zero force afterward and hence not curves such as those in Fig. 4*C*). These forces are integral multiples of 0.17 nN (see Fig. 4*F*), supporting the notion that events of type 3 (∼0.17 nN) correspond to single molecules of FH being detached, whereas events of type 2 (between ∼0.34 and ∼0.68 nN) correspond to between two and four FH molecules becoming detached from one or more C3b molecules.

Some complexes break apart cleanly, but in other complexes, stretching of molecules occurs prior to detachment (Fig. 4*E*). Indeed, the distances over which putative single pull-off events occurred range from a few nm to ∼30 nm (Fig. 4*G*). In parallel experiments, we amine-coupled FH to the AFM tip (Fig. 4*A*, *right*) instead of using a PspCN anchor, and we observed detachments occurring over distances up to 60 nm (Fig. 4*G*). Such differences in extents of elongation between modes of FH-tip attachment are expected. Firm and uniform anchoring of FH, via its central ninth CCP module (46) (of its 20 modules that are each about 4 nm in length) to PspCN would limit scope for stretching. Conversely, some amine-coupled FH molecules will be tethered to the tip via terminal regions, leaving the other terminus (modules 1–4 or modules 19–20) to bind C3b and the potential for stretching of the intervening segment of 15–17 modules (Fig. 4*A*). There is also scope for flexibility between the thioester-containing domain (TED) (binding site for CCPs 19–20) and the rest of the C3b molecule (binding site for CCPs 1–4) (Fig. 1*B*) (16, 47).

### Measuring FH:C3b and FH:C3d affinities using AFM

**Figure 5.**
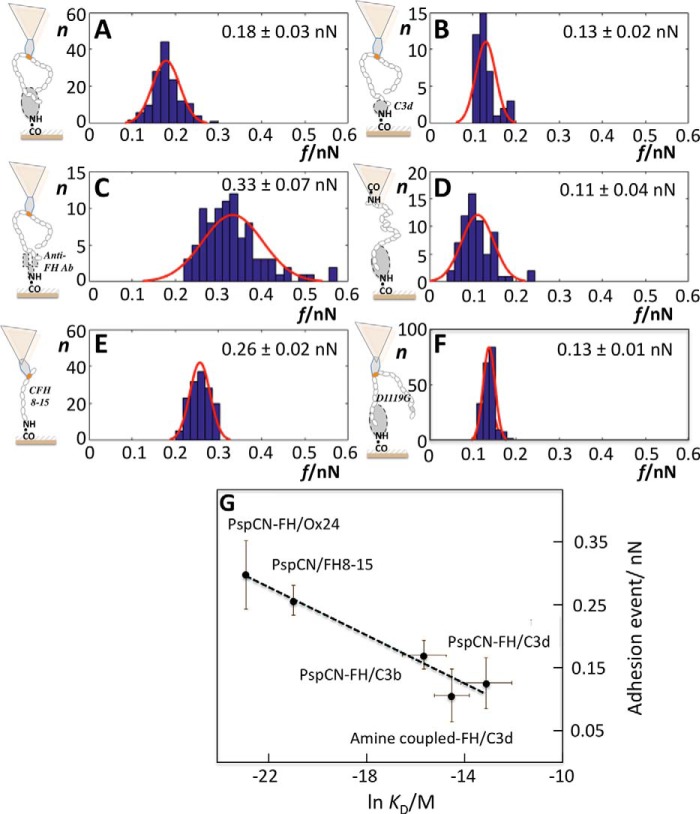
**Adhesion forces between protein molecules.** In *A–F*, AFM-derived adhesion forces (*f*) were measured, binned, and plotted for numerous individual detachment events of type 3 (see Fig. 4). The *y* axis (*n*) is the number of interactions in a bin. *A*, PspCN-anchored FH on the tip was pulled from C3b attached to the wafer. *B*, as in *A*, except C3d had been immobilized on the wafer instead of C3b. *C*, FH was amine-coupled on the tip while on the wafer was a mAb (Ox24) that recognizes CCP 5 of FH. *D*, as in *A*, except that FH was amine-coupled on the tip. *E*, interaction between PspCN (that binds CCP 9 of FH) and a truncated FH (CCPs 8–15) on the wafer. *F*, as in Fig. 5*A*, except FH was replaced with the mutant D1119G FH. *G*, the mean adhesion forces displayed in *A–F* were plotted against SPR-derived *K_D_* values. *Error bars*, S.D.

We conducted similar experiments on complexes involving FH, FH mutants, C3b, or C3d (C3d corresponds to the thioester domain of C3b and is the ultimate cleavage product of C3 *in vivo*). We focused exclusively on single-molecule (type 3) “clean-break” molecular detachments within the resulting force-distance traces to generate Fig. 5, which displays the distributions of measured adhesion forces for each AFM tip-wafer combination. Each plotted data set contains 40–200 measurements, for single-adhesion events, originating from at least two separately prepared and hence independent AFM probe tips. From these distributions, a mean ± S.D. for the force required to pull apart each complex was determined. The detachment of PspCN-anchored FH from C3d yields a narrower range of weaker forces (Fig. 5*B*) than its detachment from C3b (Fig. 5*A*). As anticipated, the complex between Ox24 (a mAb that recognizes CCPs 1–5) on the wafer and amine-coupled FH on the tip required more force to disrupt (Fig. 5*C*) than any other complex studied. A non-FH-binding antibody control showed, as expected, no measurable interaction. Amine-coupled FH was less tightly bound to C3b (Fig. 5*D*) than was PspCN-anchored FH. As expected (because it binds FH module 9), PspCN on the tip interacts with FH 8–15, a FH fragment lacking both of the primary binding sites for C3b (Fig. 5*E*), on the wafer. Fig. 5 (*A* and *F*) provides an insightful comparison as the measured pull-off force for the complex of the disease-linked CFH(D1119G) mutant with C3b is weaker (Fig. 5*F*) than the complex of the WT protein with C3b (Fig. 5*A*), and the range of values for individual complexes is narrower. All of these measured adhesion forces were plotted against the logarithm of the relevant SPR-derived *K_D_* measurements (Fig. 5*G*), revealing good correlation between these two orthogonal means of measuring interactions.

### SPR reveals surface-dependent differences in binding of CFH to C3b and C3d

All reported SPR-based studies of complement, from the first such experiments (48) to those described above, utilized sensor chips from commercial sources. However, details of commercial sensor chip manufacture and the precise chemical nature of their surfaces are proprietary information. Our success in forming SAMs on gold wafers for adhesion-force measurements led us to explore the preparation of bespoke SPR sensor chips that we called HJEM2, HJEM4, HJEM6, and HJEM7, as summarized in Table 2.

**Table 2:** **SAM components used to make four homemade sensor chips for SPR**

Chip type	Disulfide SAM I*^a^*	Disulfide SAM II	Expected SAM headgroup composition
HJEM 2	OH-COOH, 20 μm	OH-OH, 980 μm	1% COOH, 99% OH
HJEM 4	OH-COOH, 20 μm	OH-OH, 980 μm	1% COOH, 99% OH
HJEM 6	OH-propargyl, 20 μm	OH-OH, 980 μm	1% propargyl, 99% OH
HJEM 7	OH-propargyl, 500 μm	OH-COOH, 500 μm	50% OH, 25% COOH, 25% propargyl

*^a^* Each disulfide-linked undecanethiol SAM component (see supporting information and Fig. S1) is identified by the chemical natures of its two headgroups (*e.g.* OH-COOH has a hydroxyl and a carboxyl headgroup).

**Figure 6.**
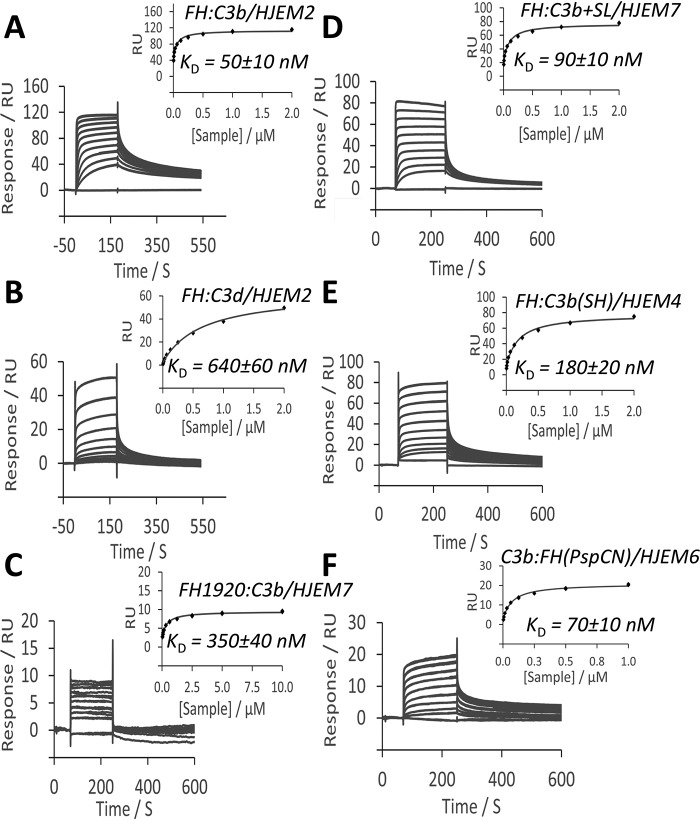
**Affinity of FH and FH 19–20 for C3b immobilized on homemade SPR sensor chips.** For details of the HJEM series of homemade sensor chips, see Table 2. For more details of experimental conditions, see Table 1. *A*, FH injected over C3b amine-coupled to HJEM2; *B*, FH injected over C3d amine-coupled to HJEM2; *C*, construct consisting of FH C-terminal domains 19 and 20 only (FH-19,20) injected over C3b amine-coupled on a HJEM7 chip. *D*, FH injected over a composite surface consisting of amine-coupled C3b and 3′-sialyllactose. *E*, FH injected over C3b thio-coupled to maleimide on a HJEM4 chip. *F*, C3b injected over PspCN-anchored FH; the PspCN had been attached via click chemistry to a HJEM6 chip.

To create HJEM2, for example, we coated a blank gold SPR chip with a SAM formed from a 99:1 ratio of hydroxyl-alkanethiols/carboxyl-alkanethiols. We used this in SPR-based measurements of FH binding to amine-coupled C3b (313 RU) (Fig. 6*A*). Unexpectedly, the *K_D_* (50 ± 10 nm) (Table 1), was 5-fold tighter than both the value obtained earlier on a C1 chip (Fig. 2*A*) and the *K_D_* measured on a commercial (C1) chip using the same C3b loading and identical reagents (not shown). A similar value of 70 ± 10 nm was obtained when FH was flowed over C3b (144 RU) amine-coupled to a chip (HJEM7; Table 2), with a higher (25%) carboxyl-group content. A homemade chip (HJEM4; Table 2) featuring maleimide groups attached to carboxyl groups allowed immobilization of hydrolyzed C3b (240 RU) via its free thiol in a quasiphysiological manner. Flowing FH over this chip (Fig. 6*E*) gave a *K_D_* (180 ± 20 nm) similar to the value reported above for physiologically coupled C3b on the C1 chip (130 ± 20 nm; Table 1). The *R*_max_ values in all of these experiments (Table 1) suggest—as observed on commercial chips—that 27–45% of immobilized C3b molecules on each HJEM chip could serve as FH-binding sites or that FH binds to two C3b molecules. The equilibrium data generally fitted less well to a 1:1 Langmuir model than those collected on commercial chips (comparing *X*^2^/*R*_max_ ratios (Table 1)), but this was not explored further, given the multitude of possible alternative modes of interaction.

Binding of FH to amine-coupled C3d (61 RU) on a HJEM2 chip fits relatively well to a 1:1 binding equation, yielding a *K_D_* of 0.64 ± 0.06 μm (Fig. 6*B*) and *R*_max_ = 61 (suggesting that ∼20% of C3d molecules are binding sites). These results contrast with the reportedly very small responses and extremely low *R*_max_ values, measured for FH binding to C3d on commercial chips (35, 49). The two C-terminal modules of FH (FH 19–20) also bound to amine-coupled C3b on our homemade chips more tightly (Fig. 6*C*) (*K_D_* = 0.35 ± 0.04 μm) compared with C3b on commercial chips (*K_D_* = 2–4 μm) (35).

We had not anticipated these large differences (*versus* commercial chips) on homemade chips that lacked embellishment with cell-surface markers such as sialic acids or glycosaminoglycans. Nonetheless, we tried making a physiologically relevant surface by preparing a sensor chip (HJEM7; Table 2) featuring propargyl headgroups that we used to click on 174 RU of 3′-sialyllactose (in the SPR instrument). As expected, flowing FH solutions over this surface (lacking C3b or C3d) gave a tiny response (not shown). On another channel, we first amine-coupled 143 RU of C3b to the chip and then clicked on 174 RU of 3′-sialyllactose. From flowing FH over this surface bearing both C3b and sialic acid, we calculated a *K_D_* = 90 ± 10 nm for the CFH:C3b interaction (Fig. 6*D*) (*versus* 70 ± 10 nm with no sialyllactose). The *R*_max_ (reflecting how many C3b molecules were available for binding) was the same (58 RUs) irrespective of 3′-sialyllactose. This implied that 3′-sialyllactose made little difference to the FH-C3b interaction.

### Is there a limit to how tightly CFH binds to C3b?

To further investigate the role of surface context on the FH:C3b interaction, we chose an SPR-based binding assay in which FH is immobilized and C3b is in solution. Using a similar strategy to that employed to anchor FH to the AFM tip described above, we captured FH (54 RU) with PspCN (18 RU) that had been clicked via an engineered-in cysteine thiol group onto propargyl groups on a HJEM6 chip (Table 2). We flowed C3b over the PspCN-anchored FH and obtained values of *K_D_* (70 nm; Fig. 6*F*) and *R*_max_ that are consistent with values for soluble FH binding to C3b immobilized on HJEM chips and 20-fold tighter than a *K_D_* reported for C3b flowed over FH that was randomly amine-coupled to a C1 chip (50).

## Discussion

We present, to the best of our knowledge, the first single-molecule images of C3b distribution following its deposition and amplification on a surface (Fig. 3*B*). The formation of clusters of C3b had previously been inferred from electron micrographs published more than 50 years ago that showed ferritin-conjugated anti-C3 antibodies on an erythrocyte physiologically decorated with C3b (32). The ferritin was distributed unevenly and included regions where 10–40 molecules occurred within 40–80 nm of cell circumference. Our AFM images feature two-dimensional clusters, in various shapes and sizes, of surface-bound C3b molecules. A lack of three-dimensional stacks or clumps shows that nascent C3b did not use its thioester to bind covalently to the nucleophilic groups of already-deposited C3b, under these conditions and at the time chosen to cease activation. This conserves—at least during the early phases of activation—access to the overlapping FB-binding and FH-binding sites on C3b.

Cluster formation is consistent with the short lifespan, relative to C3b's diffusion rate, of the exposed and activated thioester group of C3b (13). Immediately post-activation, this reacts either with surface nucleophiles or with water to form C3b(H_2_O) that diffuses away. Thus, C3b can only bind local surfaces, which is a key spatiotemporal constraint on complement activation. A quantitative study using ^131^I-C3b (18) implied that a maximum of 25 C3b molecules were deposited on Sepharose beads (a source of hydroxyl groups) for each immobilized trypsin molecule (a C3 convertase surrogate). In another experiment (51), no more than 30 C3b molecules could be deposited on zymosan (a polysaccharide prepared from yeast cell walls) per copy of C3bBb (in a scenario, unlike ours, where FB was not replenished) despite on-going C3 consumption. It was suggested that once 25 or 30 copies of C3b had clustered around a trypsin molecule, or a convertase complex, each new C3b molecule produced thereafter would be hydrolyzed before it could diffuse beyond the cluster perimeter. Using a predicted C3b-diffusion coefficient (52) = 4.5 10^−7^ cm^2^·s^−1^ and hypothetical radius of 25 nm for a circular, tightly packed cluster of 25 C3b molecules, a thioester half-life of ∼60 μs was computed by these authors and is commonly quoted.

After seven cycles of C3b amplification in our experiments, AFM revealed ∼25 clusters, of varying shapes and sizes, per μm^2^ of the chip surface, each comprised of between three and ∼20 C3b molecules. These are crowded with few gaps but not, as presumed (18), closely packed; several clusters were >100 nm across. Because we had just 25 seed C3b molecules·μm^−2^ on the chip, we conclude that most seeds had likely nucleated a cluster. It follows that most of the ∼40 isolated C3b molecules observed per μm^2^ are new, convertase-generated, molecules. Some have diffused >200 nm before touchdown, exceeding the 25-nm maximum calculated previously (see above). Using Einstein's approximation equation for two-dimensional diffusion (*t* = *x*^2^/4*D*, where *t* represents time, *D* is the diffusion constant, and *x* is distance traveled) and *D* = 4.5 10^−7^ cm^2^·s^−1^ for C3b suggests that a 200-nm journey would require nearly 250 μs. A more rigorous analysis might take into account the flow of liquid over the SPR chip (analogous to the flow of blood over a physiological surface), but we could not detect any evidence that the direction of flow had manifested itself in the shapes or distribution of the observed clusters of C3b molecules. Assuming that we can safely ignore the effects of flow, our results support a significantly longer half-life of the thioester than the 60 μs previously inferred.

In our experiments, we estimated that (after six cycles of amplification) each convertase caused just one or two C3b molecules·s^−1^ to be deposited on the surface, when fed 0.5 μm C3 (about one-tenth of the plasma C3 concentration). If, as suggested (53), one in ten C3b molecules bind to the surface, then our inferred turnover is 10–20 molecules·s^−1^ per convertase. This aligns with a kinetics study (19) that estimated *K_m_* for C3 = 5 μm and turnover (*k*_cat_) = 107 C3 molecules·s^−1^ per convertase. Thus, combining single-molecule imaging and SPR illuminates early-stage C3b amplification on surfaces. This could be explored further (*e.g.* by varying the composition of solutions injected, numbers of cycles, injection times, and surface chemistry).

Imaging the growing C3b clusters has provided fresh insights into the dynamic challenges faced by FH in controlling the potentially explosively rapid and damaging C3b-amplification process. We investigated this further by using AFM to measure the forces needed to pull apart complexes of FH and surface-immobilized C3b. Each force *versus* distance curve recorded comprised multiple interactions due to the presence of several molecules of FH on the AFM probe tip and a high density of C3b molecules on the wafer (Fig. S5). Importantly, however, individual detachment events were distinguishable, allowing force measurement for hundreds of individual complexes.

For individual complexes, “clean breaks” were observed in which the distance over which the pull off occurred was just 11–15 nm, in addition to instances where >50 nm of molecular stretching preceded separation (Fig. 4*G*). The extent of stretch thus varied between complexes but also depended on whether FH was randomly amine-coupled or PspCN-anchored specifically via its ninth module to the tip. For single, clean breaks, detachment forces ranged from 0.10 to 0.26 nN for PspCN-anchored FH, with a normal distribution, and mean = 0.17 nN, whereas mean forces measured for multiple pull-off events were integral multiples of 0.17 nN. The range of both stretching distances and, to a lesser extent, detachment forces measured in these experiments implies multiple kinds of complex forming between anchored FH and C3b attached to our artificial surface. The existence within FH of two primary C3b-binding sites (35) joined by 14 CCP modules, each ∼4 nm long (43) and linked end-to-end like beads on a string (54), is consistent with diverse conformational possibilities and variable stoichiometry. For example, one FH could bind monovalently (leaving a site unused) or bivalently to one C3b or bivalently to two C3bs. The ability of FH to adopt either “open” (binds C3b and C3d; stabilized by PspCN and some antibodies) or “closed” conformations (does not bind C3d) has been discussed (27, 34, 55, 56). In support of this model, our observations show that the FH molecule is indeed a stretchable and conformationally variable entity.

Force-distance measurements were extended to several related molecular pairings. A positive control, the FH:anti-FH antibody interaction, was the tightest, whereas the FH:C3d interaction was, as expected, the weakest (34). Between these extremes, we observed that anchoring of FH by PspCN (*versus* amine coupling of FH) increases the force needed to separate FH and C3b, supporting previously reported SPR-based comparisons (34). The D1119G mutant (57) of FH, which is linked to the kidney disease atypical hemolytic uremic syndrome and in which the C-terminal C3b-binding site is perturbed (58), was detached more easily from C3b than WT FH. Moreover, a narrower range of complexes are formed by FH(D1119G), compared with WT (Fig. 4, *A versus F*), presumably because it contains just one primary C3b-binding site (in CCPs 1–4), thus reducing the range of complexes that can form. These observations illustrate the insights offered by single-molecule approaches and suggest that these are a good way to compare the C3b-interacting properties of FH mutants.

Reassuringly, AFM-derived binding forces correlate with SPR-derived affinities (Fig. 5*G*). Thus, whereas caution is needed in analyzing whole-population measurements for FH where differences between subpopulations of distinctly different complexes are averaged away, SPR measurements remain informative. In particular, SPR delivered real-time data revealing temporal aspects of C3b amplification (Fig. 2*B*). Moreover, in SPR, analytes are flowed over ligands covalently attached to a surface, mimicking the flow of FH, FB, FD, etc. over surface-tethered C3b. On the downside, commercially available SPR sensor-chip surfaces are not designed to mimic biological surfaces. They are heavily functionalized with carboxyl groups to provide the negative charge that facilitates preconcentration of most proteins prior to immobilization and to maximize loading capacity. Crucially, it is hard to ascertain, from suppliers, modes of manufacture or precise chemical compositions of chip surface. We thus manufactured our own chips dedicated to studies of complement activation. SPR-based measurements on homemade chips yielded tighter *K_D_* values for both FH:C3b and FH:C3d than measurements on Biacore C1 or CM5 chips. This underlines the sensitivity to surfaces of FH, as a C3b binder, consistent with its surface-selective complement regulatory properties. No additional improvement in FH:C3b affinity accompanied sialic acid decoration of the homemade chip surface. This does not invalidate models in which sialic acids are important for self-surface recognition (59, 60) but demonstrates that these sugars are not needed for FH to bind tightly to surface-immobilized C3b. An “inverse” SPR-based assay, in which PspCN anchored the middle domains of FH to the chip, and soluble C3b was the analyte, implies that 50–70 nm binding of C3b is also achievable in this effectively off-surface context, provided that FH is “open” and in an appropriate conformation for binding.

By passing FH over C3b immobilized on our HJEM-series sensor chips or by flowing C3b over PspCN-anchored FH, we obtained *K_D_* values that are 5–10-fold tighter than in previous SPR-based literature. Using ^125^I-FH, Kazatchkine *et al.* (61) estimated a *K_D_* of 100 nm for FH binding to C3b on sheep erythrocytes that are regarded as selflike cells because they are protected from complement activation by human FH. On two surfaces that FH does not protect—zymosan and sheep erythrocytes treated to remove 80% of sialic acids—these authors reported a 10-fold lower affinity of FH for 80–85% of deposited C3b molecules (1.2 μm in the case of zymosan). Intriguingly, both DiScipio (also using radiolabeled FH) (51) and Kazatchkine *et al.* (61) estimated *K_D_* values in the range of 17–50 nm for a minority (15–30%) of the binding sites on C3-decorated zymosan. Thus, our values are similar to those obtained on the “selflike” surface of sheep erythrocytes (and to the stronger of the heterogeneous interactions estimated on zymosan). Conversely, the *K_D_* values of 0.5–1.5 μm obtained by us, and in numerous other reports of SPR studies performed with commercial chips (62), resemble the weaker interactions observed on nonself surfaces that are, presumably, insufficient to regulate C3b amplification. Further work is now possible to ascertain exactly which features of our homemade surfaces enhance the FH:C3b interaction and to assess the extent to which our surfaces mimic self ones in this respect. That FH binds C3d (the TED of C3b) on homemade chips (and presumably on self-surfaces *in vivo*) but not commercial ones indicates that it is the accessibility of the C terminus of FH that is critically affected by surface chemistry, as has been suggested elsewhere recently (27, 34, 56, 63).

These higher-affinity CFH:C3b interactions are compatible with multiple functional observations. Low-nm CFH is sufficient for half-maximal effects in *in vitro* DAA assays (37, 50). Similarly, both fluid-phase assays of factor I-cofactor activity and co-factor assays performed on the sheep-erythrocyte surface are generally performed with CFH at a concentration in the range of 10–20 nm (37, 50). In these *in vitro* assays of DAA and CA, FH is acting in the absence of on-going AP activation because fresh C3 is not being supplied. Notably, much higher, micromolar, levels of FH are needed to prevent erythrocyte lysis in assays using FH-depleted normal human serum (37), and indeed, FH is present at 1–2 μm in human blood. In hemolysis-protection experiments, as *in vivo*, all AP components including C3 and FB are present, and FH operates in “fire-fighting mode.” Under these circumstances, having abundant FH (∼20 times the *K_D_*) could help ensure that FH achieves blanket coverage of C3b molecules without becoming depleted or outnumbered. Significant interest has developed in potential roles of the complement system in nonvascular contexts, including in the eye (64), where levels of all complement proteins—some of which are produced locally—are orders of magnitude lower than in blood (65). Whereas the complement cascade presumably cannot operate here, FH's roles in homeostasis (7, 66) could be important, and its high-affinity interactions with C3b may be critical.

In conclusion, we have demonstrated the feasibility and the power of single-molecule level studies of the amplification of surface-bound C3b and intervention by FH. This is important because numerous diseases arise from imbalances between complement activation and regulation (9). Biological processes to ensure appropriate quantities of C3b are deposited on surfaces seem especially liable to failure, probably because the positive-feedback loop (5) driving C3b amplification magnifies the consequences of even minor functional dissimilarities between inherited variants of participating proteins, including C3, FB, FH, and FI, which are numerous and can occur in combination (67, 68). Predicting the consequences of hundreds of genetic variations requires molecular understanding, at the level discussed above, of how these proteins interact on the surfaces where complement activation and regulation compete. Such predictions would inform efforts to design, trial, and bring to market anti-complement therapeutics that have had mixed success to date (10, 69, 70).

## Experimental procedures

### Reagents

The following were purchased from Sigma-Aldrich (Merck): anhydrous 200-proof pure ethanol ≥95%, used for cleaning gold substrates and deposition of SAMs; iodoacetamide; copper (II) sulfate pentahydrate; (+)-sodium l-ascorbate; and tripotassium 5,5′,5″-[2,2′,2″-nitrilotris(methylene)tris(1*H*-benziomidazole-2,1-diyl)]tripentanoate hydrate ((BimC4A)_3_). ThermoFisher Scientific supplied streptavidin-horseradish peroxidase conjugate; *N*-(3-dimethylaminopropyl)-*N*′-ethylcarbodiimide hydrochloride (EDC); *N*-hydroxysuccinimide (NHS); and tris(2-carboxyethyl)phosphine hydrochloride (TCEP-HCl). Cysteine-HCl was from Calbiochem. *N*-(2-aminoethyl) maleimide hydrochloride was from TCI Chemicals. Azido-PEG_3_-maleimide and dibenzocyclooctyne-PEG_4_-biotin were from Jena Bioscience. The SPR amine-coupling kit and the BIAnormalizing solution (70% (w/w) glycerol) were from GE Healthcare. The SAM components, hydroxyl-PEG_6_-undecanethiol and carboxy-PEG_6_-undecanethiol, used for comparison with in-house synthesized SAM components, were purchased from Dojindo Molecular Technologies, Inc. Other SAM components were synthesized in-house (all as described in the supporting information and Fig. S4).

### Gold substrates and AFM probes

The SPR C1 sensor chips and the SIA Kit Au used to prepare our “HJEM” series of homemade sensor chips (see Table 2) were purchased from GE Healthcare. The ultra-flat gold wafers used for AFM studies were from Platypus Technologies. The AFM probes used in this study were V-shaped cantilevers with a nominal spring constant of 0.06 N/m (Cantilever D, SNL-10 Chip, Bruker). AFM probes were coated, by evaporation, with 5 nm of titanium as adhesion promoter, followed by 50 nm of gold, before chemical modification.

### Proteins

Human FH was isolated from human plasma (TCS Biosciences Ltd.), and its purity was confirmed by SDS-PAGE. Other human plasma–derived proteins (FB, FD, C3, C3b, and C3d) were purchased from Complement Technologies, Inc. (Tyler, TX). They were stored as recommended by the supplier (−80 °C) and used without further purification. The recombinant full-length FH(D1119G) mutant and recombinant FH fragments consisting of CCPs 19–20 and CCPs 8–15 were produced in *Pichia pastoris* and prepared as described (35). Recombinant sumo-Cys-PspCN was produced in *Escherichia coli* as described (34). The conjugate, sumo-Cys-PspCN-azide, was prepared by reaction of the protein (which contains one Cys residue) with azido-(PEG)_3_-maleimide following Jena Biosciences' instructions. The presence of the azide group was confirmed as follows. The suspected sumo-Cys-PspCN-azide was first reacted with iodoacetamide to block any remaining free thiol groups and then with dibenzocyclooctyne-(PEG)_4_-biotin; subsequently, the final conjugated product was detected by Western blotting with a streptavidin-horseradish peroxidase conjugate. Anti-FH mAb OX24 was from GeneTex, and goat anti-C3 polyclonal antibody was from Bioss Antibodies.

### Immobilizing C3b on C1 sensor chips for AFM studies and K_D_ measurements

A C1 sensor chip (GE Healthcare) was docked in the Biacore T200 instrument. Experiments were conducted at 25 °C. The running buffer (HBS-P) contained 10 mm HEPES buffer (pH 7.4), 150 mm NaCl, 0.05% (v/v) surfactant P20, and 1 mm MgCl_2_. Typically, one flow channel of the chip was prepared in the absence of proteins to serve as a reference (blank). C3b was amine-coupled to a second flow channel after activating the chip surface with EDC and NHS using standard procedures; to achieve the desired density of C3b molecules on the surface, a solution of 1.0 μg/ml C3b in acetate buffer, pH 5.0, was repeatedly injected at 10 μl/min until the target of 150 RUs was achieved. In a third flow channel on the chip, 18 RU of C3b (to serve as a “seed”) were amine-coupled (using a 90-s injection, 10 μl/min, of 0.5 μg/ml C3b in acetate, pH 5.0) to the surface. C3b was then physiologically coupled to the flow channel by injecting (10 μl/min) a mixture of 0.5 μm FB and 0.5 μm FD, followed by a 0.5 μm C3 injection in seven sequential cycles as follows: two cycles of FB and FD for 60 s followed by a C3 injection for 30 s, two cycles of FB and FD for 60 s followed by a C3 injection for 90 s, and three cycles of FB and FD for 60 s followed by a C3 injection for 120 s. After these seven cycles, ∼250 RUs of protein were immobilized. At a flow rate of 30 μl/min, 3 m NaCl was injected for 30 s to remove noncovalently bound proteins from the surface. After a buffer wash, the cassette was undocked, and the chip was carefully removed from the cassette for AFM. Another C1 chip, subsequently prepared in an identical way, was left in the SPR instrument and used for measurements of *K_D_* (see below).

### Preparing homemade (“HJEM-series”) SPR sensor chips

One corner of the gold surface of each new blank gold chip (GE Healthcare) was scored with a scalpel. This aided orientation of the chip in subsequent manipulations. Chips were O_2_-plasma–cleaned using a Zepto Plasma Surface Technology instrument (Diener Electronic; settings: 3 mm Hg, 40 W for 180 s). Each chip was placed in a glass vial containing ethanol, sonicated using a XUBA ultrasonic water bath (Grant) for 300 s, and rinsed with ethanol. Self-assembled monolayers were prepared by immersing the chip, immediately after cleaning, in an ethanolic solution containing a mixture of two disulfide-linked undecanethiol SAM components (of differing chemical compositions and ratios; see Fig. S4 and Table 2) at a summed total disulfide concentration of 1 mm and then incubating for 48 h at room temperature Subsequently, the SAM-coated chip surface was cleaned by three cycles of sonication in, sequentially, ethanol, ultrapure water, and ethanol again (600 s each). The chip was dried with an argon stream and stored under argon at 4 °C. For SPR studies, the chip was carefully mounted in the Biacore cassette, following the manufacturer's instructions, ensuring that the score mark used for chip orientation was concealed by the adhesive tape used to secure the chip in the cassette.

### Characterization of surfaces

Wafers/chips and AFM cantilevers (probes) were gold-coated and chemically modified using identical procedures. We therefore inferred the efficacy of our gold-coating and SAM-forming protocols on probes from the results obtained for wafers. We also characterized the Biacore-supplied blank gold-coated chips. Before SAM-coating, wafers were slightly rougher than Biacore blank chips according to AFM (1.2 *versus* 0.8-nm root mean square deviation, over a 1.0 × 1.0-μm area). This likely arose from the presence of 65-nm Au grains on our wafers compared with 40-nm grains on Biacore blanks. As expected, similar contact-angle values were measured after deposition of identical SAMs on both wafers and Biacore chips, whereas different contact-angle values were obtained for chemically different SAMs. We concluded that whether formed on wafers or blank chips, monolayers had comparable chemical compositions. Measurements by AFM showed little change in roughness after modification with SAMs and detected no contamination, indicating that surfaces remained clean despite successful modification with SAMs. Analysis of X-ray photoelectron spectroscopy yielded a composition and stoichiometry in line with the expected chemical composition of the SAM in each case. No unexpected elements were detected.

### Setting up homemade sensor chips for K_D_ determinations

A normalization procedure was required after docking each HJEM-series chip into the Biacore T200 instrument and prior to immobilizing proteins. This involved injecting a BIAnormalizing solution (70% (w/w) glycerol) to allow adjustment of the detector response and compensate for small variations in the optical system. To obtain a stable baseline, an overnight run was programmed to include five injections (30 s at 50 μl/min) of HBS-P, five of 50 mm NaOH in 1 m NaCl, five of 1 m NaCl, and finally five of 10 mm glycine, pH 2.5. This cycle of 20 injections was repeated once.

Amine coupling of C3b to the carboxyl headgroups on HJEM2 and HJEM7 chips (Table 1) was achieved using EDC/NHS-based standard techniques. For thiol coupling of C3b, a flow channel on the HJEM4 chip was activated by a 7-min injection (10 μl/min) of 0.2 m EDC and 0.05 m NHS followed by injection (10 μl/min) of *N*-2-aminoethylmaleimide (50 mm in PBS, pH 6.0) until a signal of 360 RU was obtained. The surface was then blocked by a seven-minute injection (10 μl/min) of 1 m ethanolamine, pH 8.5. A 500 μg/ml solution of pre-prepared C3b—a proportion of which contained a free thiol as a result of thioester group hydrolysis—was dissolved in PBS and injected (10 μl/min) over the flow channel until 236 RU of C3b-SH became immobilized. Remaining maleimide groups on the flow channel surface were blocked by injecting (10 μl/min) 50 mm l-Cys (in 0.1 m acetate, 1 m NaCl, pH 4.0) for 240 s, followed by a buffer wash.

In separate experiments, we sought to generate a chip surface more closely resembling host surfaces (*e.g.* those of extracellular matrix). To do this, 3′-sialyllactose was attached as follows: a click-chemistry aqueous mixture was made by sequentially adding CuSO_4_·5H_2_O (1 mm), (BimC4A)_3_ (2 mm), 3′-sialyllactose-azide (10 mm), and sodium acetate (4 mm) (all final concentrations). This mixture was incubated for 300 s and then (using the SPR instrument) injected over the HJEM7 chip (that has propargyl headgroups; see Table 1) for 30 min (1.0 μl/min). This gave 162 RU of immobilized 3′-sialyllactose. A blocking step with ethanolamine, pH 8.5 (7 min, 10 μl/min), that also washed away any nonspecifically bound 3′-sialyllactose-azide was followed by a buffer wash with 2 mm EDTA (420 s, 50 μl/min) to remove remaining Cu^2+^. A second flow channel, with a composite C3b plus 3′-sialyllactose surface, was created (in the T200 instrument) by amine-coupling C3b to the HJEM7 chip first (143 RU, using the above procedure) and then immobilizing 3′-sialyllactose-azide (174 RU) using click chemistry (see Fig. S6 for the SPR trace).

To anchor FH on a sensor chip (and subsequently employ C3b as analyte), the fusion protein sumo-Cys-PspCN-azide was reacted via a copper-catalyzed azide-alkyne 1,3-dipolar cycloaddition with propargyl groups on the chip surface. The PspCN domain is known to bind extremely tightly to FH CCPs 8–10. A similar procedure to that used for 3′-sialyllactose-azide immobilization was implemented, except 1.0 mg/ml sumo-Cys-PspCN-azide was added to the click reaction mixture, and an HJEM6 chip was used, which has 50-fold fewer propargyl groups than HJEM7 (Table 2). In total, 18 RU of PspCN were immobilized. A 80 nm solution of FH was injected over the PspCN, (30 μl/min), resulting in 54 RU of anchored FH.

### Measuring K*_D_* values by SPR

To measure the *K_D_* of the FH:C3b complex, in most cases, FH was flowed over C3b immobilized on C1 and HJEM-series chips (Table 2). For these experiments, performed in a Biacore T200 at 25 °C, a 2-fold dilution series of FH, from 0.0039 to 2 μm, all in HBS-P (pH 7.4), were injected (in duplicate, in order of increasing concentration) for 180 s at 30 μl/min, followed by a dissociation time of 500 s. Another set of measurements was performed by similarly injecting a 2-fold dilution series of the fragment FH 19–20 (the C-terminal two CCPs of FH) from 0.0039 to 10 μm. The sensor chip surface was regenerated between individual injections by either three (for the C1 chip) or six (for HJEM-series chips) 30-s 30-μl/min injections of 1 m NaCl. Baseline drift was corrected by subtracting the signal obtained from a 0 μm FH injection. Data were analyzed using the Biacore Evaluation software and a 1:1 steady-state binding model.

### Immobilization of FH on the AFM tip

For some AFM experiments, FH was immobilized on the AFM tip by exploiting its near-irreversible binding to PspCN. To achieve this, the probe was initially cleaned in ethanol and then immersed in a 1.0 mm disulfide SAM OH-maleimide (Fig. S1) for 48 h. It was subsequently rinsed sequentially in ethanol and PBS, pH 7.0, and then immersed in 100 μg/ml Cys-PspCN plus 2.5 mm tris(2-carboxyethyl)phosphine in PBS, pH 7.0. After 150 min, the probe was removed, rinsed with PBS, and then immersed in 50 mm cysteine in 0.1 m sodium acetate (pH 4.0) for 20 min to block remaining unreacted maleimide groups. After a further PBS rinse, the AFM probe was immersed in a 50 μg/ml solution of FH, PBS, pH 7.4, for 1 h prior to a final rinse with PBS, pH 7.4.

In another series of experiments, FH was amine-coupled to the AFM probe as follows. The probe was cleaned with ethanol and immersed for 48 h in a 1 mm SAM-forming solution expected to produce a monolayer with equimolar disulfide SAM OH-COOH and disulfide SAM OH-OH. The tip was subsequently removed, rinsed with ethanol and then deionized water, and immersed in 0.2 m EDC, 0.05 m NHS for 7 min. It was then rinsed in deionized water and immersed for 2 h in a solution of 30 μg/ml FH in 10 mm acetate buffer, pH 5.0. Following a further rinse in deionized water, the AFM probe was immersed in 1 m ethanolamine, pH 8.5, for 7 min to cap remaining activated esters, before a final rinse in PBS.

A mutated FH and a truncated version (FH 8–15, missing the first seven and last five of 20 CCP modules in WT FH) were amine-coupled to the probe tip in a similar way to native FH. Once prepared, FH-cantilevers were stored in PBS at 4 °C; AFM probes were always used immediately after the final rinse.

### Force measurements

Force-measurement experiments were performed in PBS using a Dimension FastScan AFM with the Icon head (Bruker). Care was taken to ensure that substrates (*i.e.* protein-decorated surfaces of gold wafers) and the protein-decorated AFM tips did not dry out during experiments. All AFM probes, substrates, and buffers to be used in an experiment were kept in the AFM hood overnight before an experiment. The laboratory housing the instrument was kept at 22 °C. Care was taken during setup and data collection to minimize temperature changes and drift.

Data were collected using the Dimension FastScan “point-and-shoot” mode, which allows the collection of force curves over several 10 × 10 grids, each grid covering an area of 1 × 1 μm. Forces were measured for grids in a minimum of five areas randomly selected from across the substrate/wafer surface. For each combination of protein on the tip and protein on the substrate reported, data were collected using a minimum of two separately prepared AFM probes.

Variations in retract speeds from 0.2 to 5 μm/s and in surface-dwell times (up to 2 s) had no significant effect on the resulting force data. A trigger point of 0.2 V, approach and retract speeds of 0.8 μm·s^−1^, and surface dwell time of 0.5 s were selected for all experiments. The same type of cantilever (SNL-10, cantilever D, Bruker) was used throughout to ensure comparability between data sets. The cantilever was calibrated at the end of each experiment to ensure that any damage to the probe from the calibration process would not hamper data collection. Calibration allowed accurate calculation of forces on the curves collected with each cantilever. Cantilever stiffness calibration was performed using the thermal tune method (71, 72). Sensitivity was measured by indentation on a nondeformable surface (freshly cleaved mica) in the measurement buffer.

### Analysis of AFM data

From each force curve collected, those pull-off events considered characteristic of unbinding events were selected for analysis. Other events were not considered. This ensured that measured adhesion values corresponded to unbinding/detachment events only, although these might involve several interacting molecules on both probe and wafer. Force curves were analyzed using a home-written MATLAB routine. The calibrated force curves allowed pull-off forces for unbinding events to be extracted from any curve that displayed such an event. Subsequently, distributions of the magnitudes of the pull-off forces, for each combination of tip protein and substrate protein, were produced, and a Gaussian distribution was fitted.

In addition to collection of force data from a given tip protein-substrate protein combination, a fresh AFM probe (ULNC-AUHW, ThermoMicroscopes, Sunnyvale, CA) with a nominal spring constant of 2.1 N/m was used to image the sample surface under the buffer. These images confirmed homogeneous coating with protein molecules. Force data were collected using intermittent contact mode over a range of scan sizes between 0.5 and 2 μm^2^. The open-source software Gwyddion (73) was used for image analysis and presentation.

## Author contributions

E. M., G. H., and P. N. B. conceptualization; E. M., R. G. B., H. J., and G. H. formal analysis; E. M., R. G. B., H. J., and J. D. P. investigation; E. M., R. G. B., H. J., J. D. P., A. N. H., and G. H. methodology; E. M., R. G. B., H. J., J. D. P., and G. H. writing-review and editing; A. N. H. and G. H. resources; A. N. H., G. H., and P. N. B. supervision; A. N. H., G. H., and P. N. B. funding acquisition; P. N. B. writing-original draft.

## Supplementary Material

Supporting Information

## Footnotes

4 The abbreviations used are:

AP: alternative pathway
AFM: atomic force microscopy
CCP: complement control protein module
FB: factor B
FD: factor D
FH: factor H
PspCN: the N-terminal domain of *Streptococcus pneumoniae* surface protein C
RU: response units
SAM: self-assembling monolayer
SPR: surface plasmon resonance
FI: factor I
N: newton(s)
(BimC4A)_3_: tripotassium 5,5′,5″-[2,2′,2″-nitrilotris(methylene)tris(1*H*-benzimidazole-2,1-diyl)]tripentanoate hydrate
EDC: *N*-(3-dimethylaminopropyl)-*N*′-ethylcarbodiimide hydrochloride
NHS: *N*-hydroxysuccinimide
TCEP: tris(2-carboxyethyl)phosphine hydrochloride
TED: thioester-containing domain.

## References

[1] Müller-Eberhard H. J. (1968) Chemistry and reaction mechanisms of complement. Adv. Immunol. 8, 1–80 10.1016/S0065-2776(08)60464-2 4174135

[2] Pangburn M. K., Schreiber R. D., and Müller-Eberhard H. J. (1983) C3b deposition during activation of the alternative complement pathway and the effect of deposition on the activating surface. J. Immunol. 131, 1930–1935 6225800

[3] Podack E. R., Esser A. F., Biesecker G., and Müller-Eberhard H. J. (1980) Membrane attack complex of complement: a structural analysis of its assembly. J. Exp. Med. 151, 301–313 10.1084/jem.151.2.301 7356725 PMC2185789

[4] Cochrane C. G., and Müller-Eberhard H. J. (1968) The derivation of two distinct anaphylatoxin activities from the third and fifth components of human complement. J. Exp. Med. 127, 371–386 10.1084/jem.127.2.371 4383923 PMC2138443

[5] Lachmann P. J. (2009) The amplification loop of the complement pathways. Adv. Immunol. 104, 115–149 10.1016/S0065-2776(08)04004-2 20457117

[6] Walport M. J. (2001) Complement: first of two parts. N. Engl. J. Med. 344, 1058–1066 10.1056/NEJM200104053441406 11287977

[7] Ricklin D., Hajishengallis G., Yang K., and Lambris J. D. (2010) Complement: a key system for immune surveillance and homeostasis. Nat. Immunol. 11, 785–797 10.1038/ni.1923 20720586 PMC2924908

[8] Ricklin D., and Lambris J. D. (2013) Complement in immune and inflammatory disorders: pathophysiological mechanisms. J. Immunol. 190, 3831–3838 10.4049/jimmunol.1203487 23564577 PMC3623009

[9] Ricklin D., Reis E. S., and Lambris J. D. (2016) Complement in disease: a defence system turning offensive. Nat. Rev. Nephrol. 12, 383–401 10.1038/nrneph.2016.70 27211870 PMC4974115

[10] Harris C. L., Pouw R. B., Kavanagh D., Sun R., and Ricklin D. (2018) Developments in anti-complement therapy; from disease to clinical trial. Mol. Immunol. 102, 89–119 10.1016/j.molimm.2018.06.008 30121124

[11] Lachmann P. J. (2018) Looking back on the alternative complement pathway. Immunobiology 223, 519–523 10.1016/j.imbio.2018.02.001 29525356

[12] Levine R. P., and Dodds A. W. (1990) The thioester bond of C3. Curr. Top. Microbiol. Immunol. 153, 73–82 10.1007/978-3-642-74977-3_4 1688756

[13] Janssen B. J., Christodoulidou A., McCarthy A., Lambris J. D., and Gros P. (2006) Structure of C3b reveals conformational changes that underlie complement activity. Nature 444, 213–216 10.1038/nature05172 17051160

[14] Pangburn M. K., and Müller-Eberhard H. J. (1980) Relation of putative thioester bond in C3 to activation of the alternative pathway and the binding of C3b to biological targets of complement. J. Exp. Med. 152, 1102–1114 10.1084/jem.152.4.1102 6903192 PMC2185963

[15] Fishelson Z., Pangburn M. K., and Müller-Eberhard H. J. (1984) Characterization of the initial C3 convertase of the alternative pathway of human complement. J. Immunol. 132, 1430–1434 6559201

[16] Chen Z. A., Pellarin R., Fischer L., Sali A., Nilges M., Barlow P. N., and Rappsilber J. (2016) Structure of complement C3(H_2_O) revealed by quantitative cross-linking/mass spectrometry and modeling. Mol. Cell Proteomics 15, 2730–2743 10.1074/mcp.M115.056473 27250206 PMC4974347

[17] Law S. K., Minich T. M., and Levine R. P. (1981) Binding reaction between the third human complement protein and small molecules. Biochemistry 20, 7457–7463 10.1021/bi00529a020 7326238

[18] Sim R. B., Twose T. M., Paterson D. S., and Sim E. (1981) The covalent-binding reaction of complement component C3. Biochem. J. 193, 115–127 10.1042/bj1930115 7305916 PMC1162583

[19] Pangburn M. K., and Müller-Eberhard H. J. (1986) The C3 convertase of the alternative pathway of human complement: enzymic properties of the bimolecular proteinase. Biochem. J. 235, 723–730 10.1042/bj2350723 3638964 PMC1146747

[20] Blatt A. Z., Pathan S., and Ferreira V. P. (2016) Properdin: a tightly regulated critical inflammatory modulator. Immunol. Rev. 274, 172–190 10.1111/imr.12466 27782331 PMC5096056

[21] Martin M., and Blom A. M. (2016) Complement in removal of the dead: balancing inflammation. Immunol. Rev. 274, 218–232 10.1111/imr.12462 27782329

[22] Kirkitadze M. D., and Barlow P. N. (2001) Structure and flexibility of the multiple domain proteins that regulate complement activation. Immunol. Rev. 180, 146–161 10.1034/j.1600-065X.2001.1800113.x 11414356

[23] Zipfel P. F., and Skerka C. (2009) Complement regulators and inhibitory proteins. Nat. Rev. Immunol. 9, 729–740 10.1038/nri2620 19730437

[24] Meri S. (2016) Self-nonself discrimination by the complement system. FEBS Lett. 590, 2418–2434 10.1002/1873-3468.12284 27393384

[25] Schmidt C. Q., Lambris J. D., and Ricklin D. (2016) Protection of host cells by complement regulators. Immunol. Rev. 274, 152–171 10.1111/imr.12475 27782321 PMC5432642

[26] Ferreira V. P., Pangburn M. K., and Cortés C. (2010) Complement control protein factor H: the good, the bad, and the inadequate. Mol. Immunol. 47, 2187–2197 10.1016/j.molimm.2010.05.007 20580090 PMC2921957

[27] Makou E., Herbert A. P., and Barlow P. N. (2013) Functional anatomy of complement factor H. Biochemistry 52, 3949–3962 10.1021/bi4003452 23701234

[28] Pangburn M. K. (2000) Host recognition and target differentiation by factor H, a regulator of the alternative pathway of complement. Immunopharmacology 49, 149–157 10.1016/S0162-3109(00)80300-8 10904114

[29] Hellwage J., Jokiranta T. S., Friese M. A., Wolk T. U., Kampen E., Zipfel P. F., and Meri S. (2002) Complement C3b/C3d and cell surface polyanions are recognized by overlapping binding sites on the most carboxyl-terminal domain of complement factor H. J. Immunol. 169, 6935–6944 10.4049/jimmunol.169.12.6935 12471127

[30] Hovingh E. S., van den Broek B., and Jongerius I. (2016) Hijacking complement regulatory proteins for bacterial immune evasion. Front. Microbiol. 7, 2004 10.3389/fmicb.2016.02004 28066340 PMC5167704

[31] Amarilyo G., Verbovetski I., Atallah M., Grau A., Wiser G., Gil O., Ben-Neriah Y., and Mevorach D. (2010) iC3b-opsonized apoptotic cells mediate a distinct anti-inflammatory response and transcriptional NF-κB-dependent blockade. Eur. J. Immunol. 40, 699–709 10.1002/eji.200838951 20039295

[32] Mardiney M. R. Jr., Müller-Eberhard H. J., and Feldman J. D. (1968) Ultrastructural localization of the third and fourth components of complement on complement-cell complexes. Am. J. Pathol. 53, 253–261 5667579 PMC2013394

[33] Oppermann M., Manuelian T., Józsi M., Brandt E., Jokiranta T. S., Heinen S., Meri S., Skerka C., Götze O., and Zipfel P. F. (2006) The C-terminus of complement regulator Factor H mediates target recognition: evidence for a compact conformation of the native protein. Clin. Exp. Immunol. 144, 342–352 10.1111/j.1365-2249.2006.03071.x 16634809 PMC1809651

[34] Herbert A. P., Makou E., Chen Z. A., Kerr H., Richards A., Rappsilber J., and Barlow P. N. (2015) Complement evasion mediated by enhancement of captured factor H: implications for protection of self-surfaces from complement. J. Immunol. 195, 4986–4998 10.4049/jimmunol.1501388 26459349 PMC4635569

[35] Schmidt C. Q., Herbert A. P., Kavanagh D., Gandy C., Fenton C. J., Blaum B. S., Lyon M., Uhrín D., and Barlow P. N. (2008) A new map of glycosaminoglycan and C3b binding sites on factor H. J. Immunol. 181, 2610–2619 10.4049/jimmunol.181.4.2610 18684951

[36] Perkins S. J., Nan R., Li K., Khan S., and Miller A. (2012) Complement Factor H-ligand interactions: self-association, multivalency and dissociation constants. Immunobiology 217, 281–297 10.1016/j.imbio.2011.10.003 22137027

[37] Kerr H., Wong E., Makou E., Yang Y., Marchbank K., Kavanagh D., Richards A., Herbert A. P., and Barlow P. N. (2017) Disease-linked mutations in factor H reveal pivotal role of cofactor activity in self-surface-selective regulation of complement activation. J. Biol. Chem. 292, 13345–13360 10.1074/jbc.M117.795088 28637873 PMC5555194

[38] Sharma A. K., and Pangburn M. K. (1996) Identification of three physically and functionally distinct binding sites for C3b in human complement factor H by deletion mutagenesis. Proc. Natl. Acad. Sci. U.S.A. 93, 10996–11001 10.1073/pnas.93.20.10996 8855297 PMC38272

[39] Jokiranta T. S., Hellwage J., Koistinen V., Zipfel P. F., and Meri S. (2000) Each of the three binding sites on complement factor H interacts with a distinct site on C3b. J. Biol. Chem. 275, 27657–27662 10.1074/jbc.M002903200 10837479

[40] Harris C. L., Abbott R. J., Smith R. A., Morgan B. P., and Lea S. M. (2005) Molecular dissection of interactions between components of the alternative pathway of complement and decay accelerating factor (CD55). J. Biol. Chem. 280, 2569–2578 10.1074/jbc.M410179200 15536079

[41] Dave S., Pangburn M. K., Pruitt C., and McDaniel L. S. (2004) Interaction of human factor H with PspC of *Streptococcus pneumoniae*. Indian J. Med. Res. 119, 66–73 15232165

[42] Soares D. C., and Barlow P. N. (2005) Complement control protein modules in the regulators of complement activation. in Structural Biology of the Complement System (Morikis D., and Lambris J. D., eds) pp. 19–62, CRC Press, Inc., Boca Raton, FL

[43] Norman D. G., Barlow P. N., Baron M., Day A. J., Sim R. B., and Campbell I. D. (1991) Three-dimensional structure of a complement control protein module in solution. J. Mol. Biol. 219, 717–725 10.1016/0022-2836(91)90666-T 1829116

[44] Schmidt C. Q., Herbert A. P., Mertens H. D., Guariento M., Soares D. C., Uhrin D., Rowe A. J., Svergun D. I., and Barlow P. N. (2010) The central portion of factor H (modules 10–15) is compact and contains a structurally deviant CCP module. J. Mol. Biol. 395, 105–122 10.1016/j.jmb.2009.10.010 19835885 PMC2806952

[45] Makou E., Mertens H. D., Maciejewski M., Soares D. C., Matis I., Schmidt C. Q., Herbert A. P., Svergun D. I., and Barlow P. N. (2012) Solution structure of CCP modules 10–12 illuminates functional architecture of the complement regulator, factor H. J. Mol. Biol. 424, 295–312 10.1016/j.jmb.2012.09.013 23017427 PMC4068365

[46] Achila D., Liu A., Banerjee R., Li Y., Martinez-Hackert E., Zhang J. R., and Yan H. (2015) Structural determinants of host specificity of complement factor H recruitment by *Streptococcus pneumoniae*. Biochem. J. 465, 325–335 10.1042/BJ20141069 25330773 PMC5600146

[47] Nishida N., Walz T., and Springer T. A. (2006) Structural transitions of complement component C3 and its activation products. Proc. Natl. Acad. Sci. U.S.A. 103, 19737–19742 10.1073/pnas.0609791104 17172439 PMC1750921

[48] Jokiranta T. S., Westin J., Nilsson U. R., Nilsson B., Hellwage J., Löfås S., Gordon D. L., Ekdahl K. N., and Meri S. (2001) Complement C3b interactions studied with surface plasmon resonance technique. Int. Immunopharmacol. 1, 495–506 10.1016/S1567-5769(00)00042-4 11367533

[49] Morgan H. P., Schmidt C. Q., Guariento M., Blaum B. S., Gillespie D., Herbert A. P., Kavanagh D., Mertens H. D., Svergun D. I., Johansson C. M., Uhrín D., Barlow P. N., and Hannan J. P. (2011) Structural basis for engagement by complement factor H of C3b on a self surface. Nat. Struct. Mol. Biol. 18, 463–470 10.1038/nsmb.2018 21317894 PMC3512577

[50] Tortajada A., Montes T., Martínez-Barricarte R., Morgan B. P., Harris C. L., and de Córdoba S. R. (2009) The disease-protective complement factor H allotypic variant Ile62 shows increased binding affinity for C3b and enhanced cofactor activity. Hum. Mol. Genet. 18, 3452–3461 10.1093/hmg/ddp289 19549636 PMC3272369

[51] DiScipio R. G. (1981) The binding of human complement proteins C5, factor B, β 1H and properdin to complement fragment C3b on zymosan. Biochem. J. 199, 485–496 10.1042/bj1990485 6462133 PMC1163402

[52] Tack B. D., and Prahl J. W. (1976) Third component of human complement: purification from plasma and physicochemical characterization. Biochemistry 15, 4513–4521 10.1021/bi00665a028 823964

[53] Müller-Eberhard H. J., Polley M. J., and Calcott M. A. (1967) Formation and functional significance of a molecular complex derived from the second and the fourth component of human complement. J. Exp. Med. 125, 359–380 10.1084/jem.125.2.359 6019133 PMC2138355

[54] Barlow P. N., Steinkasserer A., Norman D. G., Kieffer B., Wiles A. P., Sim R. B., and Campbell I. D. (1993) Solution structure of a pair of complement modules by nuclear magnetic resonance. J. Mol. Biol. 232, 268–284 10.1006/jmbi.1993.1381 8331663

[55] Osborne A. J., Nan R., Miller A., Bhatt J. S., Gor J., and Perkins S. J. (2018) Two distinct conformations of factor H regulate discrete complement-binding functions in the fluid phase and at cell surfaces. J. Biol. Chem. 293, 17166–17187 10.1074/jbc.RA118.004767 30217822 PMC6222095

[56] Pouw R. B., Brouwer M. C., de Gast M., van Beek A. E., van den Heuvel L. P., Schmidt C. Q., van der Ende A., Sánchez-Corral P., Kuijpers T. W., and Wouters D. (2019) Potentiation of complement regulator factor H protects human endothelial cells from complement attack in aHUS sera. Blood Adv. 3, 621–632 10.1182/bloodadvances.2018025692 30804016 PMC6391659

[57] Richards A., Buddles M. R., Donne R. L., Kaplan B. S., Kirk E., Venning M. C., Tielemans C. L., Goodship J. A., and Goodship T. H. (2001) Factor H mutations in hemolytic uremic syndrome cluster in exons 18–20, a domain important for host cell recognition. Am. J. Hum. Genet. 68, 485–490 10.1086/318203 11170896 PMC1235281

[58] Morgan H. P., Mertens H. D., Guariento M., Schmidt C. Q., Soares D. C., Svergun D. I., Herbert A. P., Barlow P. N., and Hannan J. P. (2012) Structural analysis of the C-terminal region (modules 18–20) of complement regulator factor H (FH). PLoS One 7, e32187 10.1371/journal.pone.0032187 22389686 PMC3289644

[59] Meri S., and Pangburn M. K. (1990) Discrimination between activators and nonactivators of the alternative pathway of complement: regulation via a sialic acid/polyanion binding site on factor H. Proc. Natl. Acad. Sci. U.S.A. 87, 3982–3986 10.1073/pnas.87.10.3982 1692629 PMC54028

[60] Pangburn M. K. (2002) Cutting edge: localization of the host recognition functions of complement factor H at the carboxyl-terminal: implications for hemolytic uremic syndrome. J. Immunol. 169, 4702–4706 10.4049/jimmunol.169.9.4702 12391176

[61] Kazatchkine M. D., Fearon D. T., and Austen K. F. (1979) Human alternative complement pathway: membrane-associated sialic acid regulates the competition between B and β1 H for cell-bound C3b. J. Immunol. 122, 75–81 762425

[62] Perkins S. J., Nan R., Okemefuna A. I., Li K., Khan S., and Miller A. (2010) Multiple interactions of complement Factor H with its ligands in solution: a progress report. Adv. Exp. Med. Biol. 703, 25–47 10.1007/978-1-4419-5635-4_3 20711705

[63] Harder M. J., Anliker M., Höchsmann B., Simmet T., Huber-Lang M., Schrezenmeier H., Ricklin D., Lambris J. D., Barlow P. N., and Schmidt C. Q. (2016) Comparative analysis of novel complement-targeted inhibitors, MiniFH, and the natural regulators factor H and factor H-like protein 1 reveal functional determinants of complement regulation. J. Immunol. 196, 866–876 10.4049/jimmunol.1501919 26643478 PMC4707092

[64] Warwick A., Khandhadia S., Ennis S., and Lotery A. (2014) Age-related macular degeneration: a disease of systemic or local complement dysregulation? J. Clin. Med. 3, 1234–1257 10.3390/jcm3041234 26237601 PMC4470180

[65] Loyet K. M., Deforge L. E., Katschke K. J. Jr., Diehl L., Graham R. R., Pao L., Sturgeon L., Lewin-Koh S. C., Hollyfield J. G., and van Lookeren Campagne M. (2012) Activation of the alternative complement pathway in vitreous is controlled by genetics in age-related macular degeneration. Invest. Ophthalmol. Vis. Sci. 53, 6628–6637 10.1167/iovs.12-9587 22930722

[66] Trouw L. A., Bengtsson A. A., Gelderman K. A., Dahlbäck B., Sturfelt G., and Blom A. M. (2007) C4b-binding protein and factor H compensate for the loss of membrane-bound complement inhibitors to protect apoptotic cells against excessive complement attack. J. Biol. Chem. 282, 28540–28548 10.1074/jbc.M704354200 17699521

[67] Harris C. L., Heurich M., Rodriguez de Cordoba S., and Morgan B. P. (2012) The complotype: dictating risk for inflammation and infection. Trends Immunol. 33, 513–521 10.1016/j.it.2012.06.001 22749446 PMC3460238

[68] Goicoechea de Jorge E., López Lera A., Bayarri-Olmos R., Yebenes H., Lopez-Trascasa M., and Rodríguez de Córdoba S. (2018) Common and rare genetic variants of complement components in human disease. Mol. Immunol. 102, 42–57 10.1016/j.molimm.2018.06.011 29914697

[69] Kassa E., Ciulla T. A., Hussain R. M., and Dugel P. U. (2019) Complement inhibition as a therapeutic strategy in retinal disorders. Expert Opin. Biol. Ther. 19, 335–342 10.1080/14712598.2019.1575358 30686077

[70] Park D. H., Connor K. M., and Lambris J. D. (2019) The challenges and promise of complement therapeutics for ocular diseases. Front. Immunol. 10, 1007 10.3389/fimmu.2019.01007 31156618 PMC6529562

[71] Hutter J., and Bechhoefer J. (1993) Calibration of atomic-force microscope tips. Rev. Sci. Instrum. 64, 1868–1873 10.1063/1.1143970

[72] Butt H.-J., and Jaschke M. (1995) Calculation of thermal noise in atomic force microscopy. Nanotechnology 6, 1–7 10.1088/0957-4484/6/1/001

[73] Nečas D., and Klapetek P. (2012) Gwyddion: an open-source software for SPM data analysis. Cent. Eur. J. Phys. 10, 181–188 10.2478/s11534-011-0096-2

