# Supplementary material for: Combining SPR with atomic-force microscopy enables single-molecule insights into activation and suppression of the complement cascade
Source: J Biol Chem. 2019 Nov 12;294(52):20148–63. doi: 10.1074/jbc.RA119.010913 (PMC6937562; doi:10.1074/jbc.RA119.010913)
Supplement: Supporting Information [file supp_294_52_20148__index.html]

Combining SPR with atomic-force microscopy enables single-molecule insights into activation and suppression of the complement cascade — Single-molecule studies of complement factor H — Combining SPR with atomic-force microscopy enables single-molecule insights into activation and suppression of the complement cascade — Single-molecule studies of complement regulation — Supporting Information 

# Combining SPR with atomic-force microscopy enables single-molecule insights into activation and suppression of the complement cascade

## Supporting Information

- Supporting Information (to be published online) - Details of chemical synthesis and other additional figures
