# Supplementary material for: Combining SPR with atomic-force microscopy enables single-molecule insights into activation and suppression of the complement cascade
Source: J Biol Chem. 2019 Nov 12;294(52):20148–63. doi: 10.1074/jbc.RA119.010913 (PMC6937562; doi:10.1074/jbc.RA119.010913)
Supplement: Supporting Information [file supp_RA119.010913_155603_2_supp_428047_q0v6lx.pdf]

Combining SPR with AFM enables single-molecule insights into activation and suppression of the complement cascade

Elisavet Makou, Richard G Bailey, Heather Johnston, John D Parkin, Alison Hulme, Georg Hähner and Paul N Barlow

Supplementary information

CONTENTS

Synthesis of SAM components

Supplementary Figures

Supplementary Figure1 *Counting C3b molecules on a C1 sensor chip*
Supplementary Figure2 *Representative SPR trace for physiological immobilisation of C3b on a C1 chip*
Supplementary Figure3 *Counting molecules within clusters.*
Supplementary Figure4 *Chemical structures of SAM components*
Supplementary Figure5 *AFM-derived images of wafer and cantilever (tip) surfaces used in force- distance measurements.*
Supplementary Figure6 *Preparation of the 3'-sialyllactose- and C3b-decorated HJEM 7 surface*

Synthesis of SAM components

General Synthetic Methods

All non-aqueous reactions were carried out under an atmosphere of nitrogen using oven-dried glassware that was cooled in a desiccator prior to use. Unless otherwise noted, starting materials and reagents were obtained from commercial suppliers and were used without further purification. Toluene, THF, CH_2Cl_2, and Et_2O were dried and purified by passage through activated alumina columns using a Glass Contour Solvent Purification System. Saturated aqueous solutions of inorganic salts are represented as (volume, sat aq). ^1H and ^{13}C NMR spectra were obtained on Bruker instruments at the stated frequency. Infra-red spectra were recorded neat on Shimadzu IRAffinity-1 unless otherwise stated. Electrospray (ESI) and electron ionisation (EI) mass spectra were obtained on a Kratos MS50TC mass spectrometer. Melting points were determined on a Gallenkamp Electrothermal Melting Point apparatus and are uncorrected. Flash chromatography was carried out using Merck Kieselgel 60 (Merck 9385) under positive pressure. Eluent compositions are quoted as v/v ratios.

General Synthetic Scheme

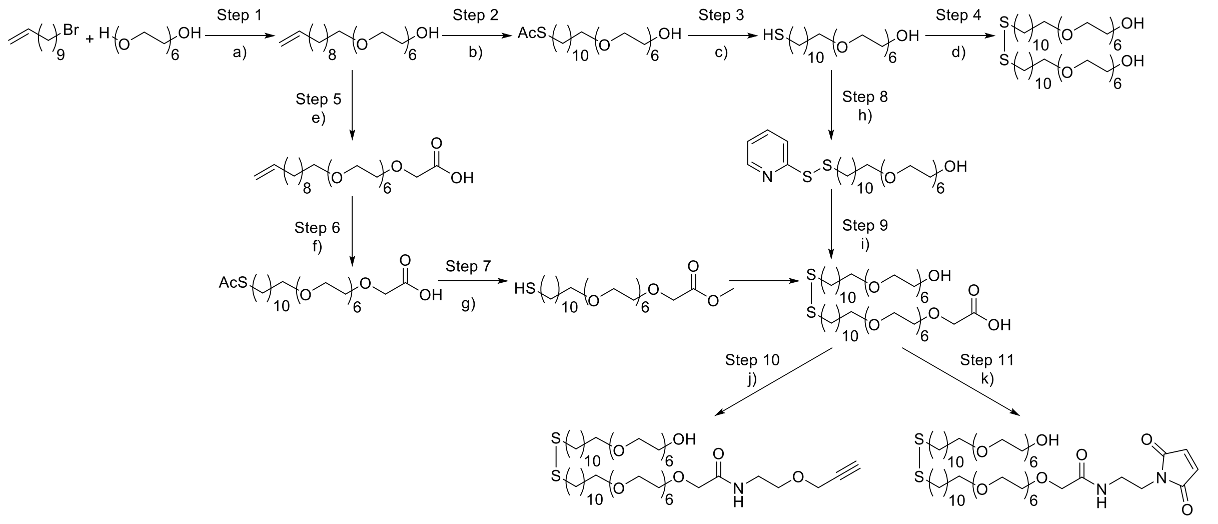

Reagents and conditions: a) NaH, DMF, 0 °C - rt, 16 h, 71%; b) Thioacetic acid, AIBN, THF, hυ, rt, 3 h, 86%; c) HCl, MeOH, 110 °C, 2.5 h, 93%; d) I_2, MeOH, rt, 2h, 38%; e) Bromoacetic acid, NaH, DMF, 0 °C - rt, 16h, Quant.; f) Thioacetic acid, AIBN, THF, hυ, rt, 3 h, 89%; g) Acetyl chloride, MeOH, reflux, 3 h, Quant.; h) Aldrithiol-2, NEt_3, MeOH, rt, 16 h, 84%; i) DMAP, THF, rt, 8 h, 42%, j) 2-(prop-2-yn-1-yloxy)ethan-1-amine, DIC, Oxyma, DIPEA, DCM, rt 2 h, 39%.

Synthesis of SAM precursors

Step 1: Synthesis of 3,6,9,12,15,18-hexaoxanonacos-28-en-1-ol

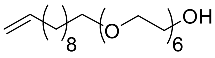

Sodium hydride (0.21 g, 5.4 mmol, 60% in mineral oil) was added in small portions to a stirred solution of hexaethylene glycol (1.1 mL, 4.2 mmol) in dry DMF (10 mL) at rt. The reaction mixture was stirred for 30 min at rt then cooled to 0 °C and 11-bromo-1-undecene (0.47 mL, 2.1 mmol) in dry DMF (2.5 mL) was added dropwise. The reaction mixture was allowed to return to rt then stirred for ~16 h. NH_4Cl (8 mL, sat. aq.) was added dropwise followed by EtOAc (25 mL). The organic layer was separated then the aqueous layer was further extracted with ethyl acetate (3 × 25 mL). The combined organic layers were washed with brine (2 × 25 mL) then dried with anhydrous sodium sulfate. The crude product was concentrated *in vacuo,* freeze dried to remove DMF, then purified by column chromatography (DCM – DCM/MeOH, 98:2) to give the desired product as a pale yellow oil (0.66 g, 71 %). **R_f** (DCM:MeOH, 19:1) = 0.39; **IR** (neat, cm^{-1}) 3650 – 3250 (O–H), 1639 (C=C), 1105 (C–O); **^{1}H NMR** δ (601 MHz, $CDCl_3$) 5.81 (1H, ddt, *J* = 16.9, 10.2, 6.6 Hz, C*H*=CH_2), 5.02 – 4.89 (2H, m, CH=CH_2), 3.74 – 3.56 (24H, m, 6 × OCH_2CH_2O), 3.44 (2H, t, *J* = 6.9 Hz, CH_2O), 2.15 (1H, br s, O*H*), 2.06 – 2.01 (2H, m, CH_2CH=CH_2), 1.57 (2H, p, *J* = 6.9 Hz, CH_2), 1.41 – 1.22 (12H, m, 6 × CH_2); **^{13}C NMR** δ (151 MHz, $CDCl_3$) 139.38 (CH), 114.24 (CH_2), 72.69 (CH_2), 71.70 (CH_2), 70.79 (CH_2), 70.74 (6 × CH_2), 70.71 (CH_2), 70.51 (CH_2), 70.21 (CH_2), 61.92 (CH_2), 33.95 (CH_2), 29.79 (CH_2), 29.69 (CH_2), 29.62 (CH_2), 29.58 (CH_2), 29.27 (CH_2), 29.08 (CH_2), 26.24 (CH_2); ***m/z*** (ESI+, MeOH) 457 ($[M+Na]^+$, 100%).

Step 2: Synthesis of 1-hydroxy-3,6,9,12,15,18-hexaoxa-30-thiadotriacontan-31-one

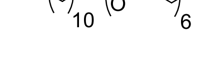

Thioacetic acid (0.30 mL, 4.1 mmol) and AIBN (0.18 g, 1.1 mmol) were added to a stirred solution of 3,6,9,12,15,18-hexaoxanonacos-28-en-1-ol (0.61 g, 1.4 mmol) in dry, degassed THF (10 mL) and the mixture was irradiated with UV light for 3h at rt. The reaction mixture was concentrated *in vacuo* and the crude product was purified by column chromatography (DCM – DCM/MeOH, 98:2) to give the desired product as a colourless oil (0.61 g, 86%). **R_f** (DCM:MeOH, 19:1) = 0.47; **IR** (neat, cm^{-1}) 1692 (C=O), 1101 (C–O); **^{1}H NMR** δ (601 MHz, $CDCl_3$) 3.74 – 3.50 (24H, m, 6 × OCH_2CH_2O), 3.42 (2H, t, *J* = 6.8 Hz, CH_2O), 2.87 – 2.80 (m, 2H, CH_2), 2.30 (s, 3H, COCH_3), 1.60 – 1.48 (m, 4H, 2 × CH_2), 1.38 – 1.17 (m, 14H, 7 × CH_2) (OH proton not

observed); **^{13}C NMR** δ (151 MHz, $CDCl_3$) 196.12 (C), 77.37 (CH_2), 77.16 (CH_2), 76.95 (CH_2), 72.69 (CH_2), 71.63 (CH_2), 70.72 (CH_2), 70.68 (3 × CH_2), 70.64 (CH_2), 70.43 (CH_2), 70.15 (CH_2), 61.83 (CH_2), 30.73 (CH_3), 29.73 (CH_2), 29.65 (CH_2), 29.60 (2 × CH_2), 29.57 (CH_2), 29.55 (CH_2), 29.26 (CH_2), 29.20 (CH_2), 28.91 (CH_2), 26.18 (CH_2); ***m/z*** (ESI+, MeOH) 533 ($[M+Na]^+$, 100%), 528 (65).

Step 3: Synthesis of 29-sulfanyl-3,6,9,12,15,18-hexaoxanonacosan-1-ol

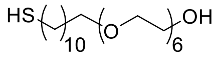

1-Hydroxy-3,6,9,12,15,18-hexaoxa-30-thiadotriacontan-31-one (3.1 g, 6.1 mmol) was dissolved in MeOH (30 mL) and HCl (10 mL, 3.5 M Aq) and refluxed at 100 ºC for 3 h. The reaction was allowed to cool to rt, then DCM (100 mL) was added and the organic layer was separated. The organic material was washed with H_2O (3 × 30 mL) and $NaHCO_3$ (3 × 30 mL, Sat Aq), dried with Na_2SO_4 and concentrated *in vacuo* to give the desired product as a yellow oil (2.8 g, 98%) which was used in the next reaction without further purification. **R_f** (DCM:MeOH, 19:1) = 0.34; **IR** (neat, cm^{-1}) 3600 – 3200 (O–H), 1109 (C–O); **^{1}H NMR** δ (601 MHz, $CDCl_3$) δ 3.79 – 3.57 (24H, m, 6 × OCH_2CH_2O), 3.47 (2H, t, *J* = 6.8 Hz, CH_2), 2.54 (2H, dd, *J* = 14.7, 7.5 Hz, CH_2), 1.95 (1H, br s, OH), 1.66 – 1.55 (4H, m, 2 × CH_2), 1.44 – 1.25 (15H, m, 7 × CH_2, 1 × SH); **^{13}C NMR** δ (151 MHz, $CDCl_3$) δ 72.57 (CH_2), 71.54 (CH_2), 70.63 (CH_2), 70.58 (6 × CH_2), 70.55 (CH_2), 70.34 (CH_2), 70.05 (CH_2), 61.76 (CH_2), 34.05 (CH_2), 29.64 (CH_2), 29.56 (CH_2), 29.51 (CH_2), 29.49 (CH_2), 29.47 (CH_2), 29.06 (CH_2), 28.37 (CH_2), 26.09 (CH_2), 24.65 (CH_2); ***m/z*** (ESI+, MeOH) 486 ($[M+NH_4]^+$, 100).

Step 4: Synthesis of 3,6,9,12,15,18,43,46,49,52,55,58-dodecaoxa-30,31-dithiahexacontane-1,60-diol ("Disulfide SAM OH-OH")

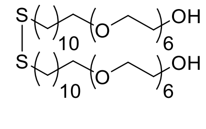

Iodine (0.15 g, 0.59 mmol) was added to a stirred solution of 29-sulfanyl-3,6,9,12,15,18-hexaoxanonacosan-1-ol (0.55 g, 1.2 mmol) in MeOH (12 mL) and the reaction was stirred at rt for 2 h. The reaction mixture was concentrated *in vacuo* then redissolved in EtOAc (40 mL). The organic solution was washed with $Na_2S_2O_4$ (2 × 30 mL, 10% Aq) and brine (1 × 30 mL), dried with Na2SO4 and concentrated *in vacuo*. The crude material was purified by column chromatography (DCM – DCM/MeOH, 98:2) to give the desired product as a colourless oil which solidified on standing (0.21 g, 38%). **R_f** (DCM:MeOH, 19:1) = 0.32; **IR** (neat, cm^{-1}) 3700 – 3300

(O–H), 1101 (C–O); **^{1}H NMR** δ (601 MHz, $CDCl_3$) 3.77 – 3.56 (48H, m, 12 × OCH_2CH_2O), 3.46 (4H, t, *J* = 6.8 Hz, 2 × CH_2), 2.73 – 2.65 (4H, m, 2 × CH_2), 1.68 (4H, dt, *J* = 14.9, 7.4 Hz, 2 × CH_2), 1.62 – 1.54 (4H, m, 2 × CH_2), 1.42 – 1.25 (28H, m, 14 × CH_2) (2 × OH proton not observed); **^{13}C NMR** δ (151 MHz, $CDCl_3$) 72.56 (2 × CH_2), 71.54 (2 × CH_2), 70.62 (2 × CH_2), 70.58 (2 × CH_2), 70.55 (12 × CH_2), 70.34 (2 × CH_2), 70.05 (2 × CH_2), 61.74 (2 × CH_2), 39.19 (2 × CH_2), 29.64 (2 × CH_2), 29.57 (2 × CH_2), 29.53 (2 × CH_2), 29.50 (2 × CH_2), 29.48 (2 × CH_2), 29.24 (2 × CH_2), 29.23 (2 × CH_2), 28.53 (2 × CH_2), 26.09 (2 × CH_2); ***m/z*** (ESI+, MeOH) 958 ($[M+Na]^+$, 50%), 487 (100).

Step 5: Synthesis of 3,6,9,12,15,18,21-heptaoxadotriacont-31-enoic acid

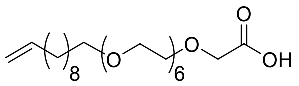

A solution of 3,6,9,12,15,18-hexaoxanonacos-28-en-1-ol (3.78 g, 8.70 mmol) in THF (20 mL) was added dropwise to a stirred suspension of sodium hydride (1.17 g, 30.4 mmol, 60% in mineral oil) in THF (15 mL). After 30 min a solution of bromoacetic acid (1.81 g, 13.0 mmol) in THF (15 mL) was added dropwise and the reaction was heated to 60 °C for ~16 h. The reaction was allowed to cool to rt, then was quenched by addition of HCl (50 mL, 1M Aq). The organic solvent was removed *in vacuo* and the resulting aqueous material was extracted with Et_2O (5 × 50 mL). The combined organic layers were washed with HCl (4 × 50 mL, 1M Aq) and brine (2 × 50 mL), then dried over $MgSO_4$ and concentrated *in vacuo* to give the desired product as a yellow oil (4.30 g, Quant.) which was used in the next reaction without further purification. **R_f** (DCM:MeOH, 19:1) = 0.15; **IR** (neat, cm^{-1}) 2950 – 2800 (O–H), 1740 (C=O), 1639 (C=C), 1105 (C–O); **^{1}H NMR** δ (601 MHz, $CDCl_3$) 5.81 (1H, ddt, *J* = 17.0, 10.2, 6.7 Hz, $CH_2CH=CH_2$), 5.06 – 4.87 (2H, m, $CH_2CH=CH_2$), 4.16 (2H, s, CH_2COOH), 3.83 – 3.52 (24H, m, OCH_2CH_2O), 3.45 (2H, t, *J* = 6.8 Hz, $CH_2CH_2CH_2$O), 2.09 – 1.96 (2H, m, $CH_2CH=CH_2$), 1.63 – 1.51 (2H, m, CH_2CH_2), 1.42 – 1.16 (12H, m, 6 × CH_2CH_2) (OH proton not observed); **^{13}C NMR** δ (151 MHz, $CDCl_3$) 171.59 (C), 139.39 (CH), 114.24 (CH_2), 71.70 (CH_2), 71.41 (CH_2), 70.81 (CH_2), 70.80 (CH_2), 70.71 (CH_2), 70.69 (CH_2), 70.68 (2 × CH_2), 70.63 (CH_2), 70.59 (CH_2), 70.55 (CH_2), 70.45 (CH_2), 70.17 (CH_2), 69.38 (CH_2), 33.96 (CH_2), 29.74 (CH_2), 29.69 (CH_2), 29.62 (CH_2), 29.59 (CH_2), 29.28 (CH_2), 29.09 (CH_2), 26.22 (CH_2); ***m/z*** (ESI+, MeOH) 515 ($[M+Na]^+$, 58%), 510 ($[M+NH_4]^+$, 100).

Step 6: Synthesis of 34-oxo-3,6,9,12,15,18,21-heptaoxa-33-thiapentatriacontanoic acid

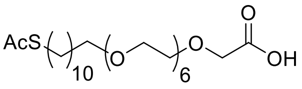

Thioacetic acid (1.87 mL, 26.2 mmol) and AIBN (1.15 g, 6.98 mmol) were added to a stirred solution of 3,6,9,12,15,18,21-heptaoxadotriacont-31-enoic acid (4.30 g, 8.73 mmol) in dry, degassed THF (50 mL) and the mixture was irradiated with UV light for 3 h at rt. The reaction mixture was concentrated *in vacuo* and the crude product was purified by column chromatography (DCM – DCM/MeOH, 98:2) to give the desired product as a colourless oil (4.43 g, 89%). $\mathbf{R_f}$ (DCM:MeOH, 19:1) = 0.31; **IR** (neat, cm^{-1}) 3000 – 2800 (O–H), 1740 (C=O), 1692 (C=O), 1107 (C–O); ^{1}**H NMR** δ (601 MHz, $CDCl_3$) 4.13 (2H, s, CH_2COOH), 3.77 – 3.54 (24H, m, 6 × OCH_2CH_2O), 3.44 (2H, t, *J* = 6.8 Hz, CH_2CH_2CH_2O), 2.86 (2H, t, *J* = 7.4 Hz, 2H, CH_2SCOCH_3), 2.32 (3H, s, SCOCH_3), 1.56 (4H, tt, *J* = 8.3, 6.5 Hz, 2 × CH_2CH_2), 1.37 – 1.23 (14H, m, 7 × CH_2CH_2) (OH proton not observed); ^{13}**C NMR** δ (151 MHz, $CDCl_3$) 196.22 (C), 172.35 (C), 71.68 (CH_2), 71.10 (CH_2), 70.64 (CH_2), 70.62 (CH_2), 70.58 (2 × CH_2), 70.54 (CH_2), 70.53 (CH_2), 70.47 (3 × CH_2), 70.32 (CH_2), 70.14 (CH_2), 69.52 (CH_2), 30.79 (CH_3), 29.75 (CH_2), 29.71 (CH_2), 29.66 (CH_2), 29.65 (CH_2), 29.63 (CH_2), 29.61 (CH_2), 29.32 (CH_2), 29.26 (CH_2), 28.97 (CH_2), 26.23 (CH_2); ***m/z*** (ESI+, MeOH) 591 ($[M+Na]^+$, 100%), 586 (50).

Step 7: Synthesis of methyl 32-mercapto-3,6,9,12,15,18,21-heptaoxadotriacontanoate

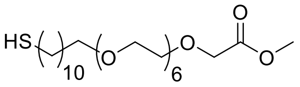

Acetyl chloride (1.66 mL, 23.4 mmol) was added dropwise to a stirred solution of MeOH (30 mL) at 0 °C and the reaction was stirred for 15 mins. 34-Oxo-3,6,9,12,15,18,21-heptaoxa-33-thiapentatriacontanoic acid (4.43 g, 7.80 mmol) in MeOH (20 mL) was added dropwise and the reaction was heated to reflux for 3 h. The reaction was allowed to cool to rt then was concentrated *in vacuo* to give the desired product as a yellow oil (4.38 g, Quant.) which was used in the next reaction without further purification. **IR** (neat, cm^{-1}) 1760 (C=O), 1100 (C–O); ^{1}**H NMR** δ (601 MHz, $CDCl_3$) 4.17 (2H, s, CH_2COOCH_3), 3.75 (3H, s, COOCH_3), 3.74 – 3.54 (24H, m, 6 × OCH_2CH_2O), 3.44 (2H, t, *J* = 6.8 Hz, CH_2CH_2CH_2O), 2.52 (2H, q, *J* = 7.4 Hz, CH_2SH), 1.69 – 1.50 (4H, m, 2 × CH_2CH_2), 1.42 – 1.20 (14H, m, 7 × CH_2CH_2) (SH proton not observed); ^{13}**C NMR** δ (151 MHz, $CDCl_3$) 170.90 (C), 71.56 (CH_2), 70.98 (CH_2), 70.68 (CH_2), 70.65 (2 ×CH_2), 70.62 (6 × CH_2), 70.56 (CH_2), 70.07 (CH_2), 68.71

(CH_2), 51.84 (CH_3), 34.05 (CH_2), 29.65 (CH_2), 29.56 (CH_2), 29.52 (CH_2), 29.50 (CH_2), 29.48 (CH_2), 29.07 (CH_2), 28.38 (CH_2), 26.10 (CH_2), 24.66 (CH_2); ***m/z*** (ESI+, MeOH) 541 ($[M+H]^+$, 100%).

Step 8: Synthesis of 1-(pyridin-2-yl)-14,17,20,23,26,29-hexaoxa-1,2-dithiahentriacontan-31-ol

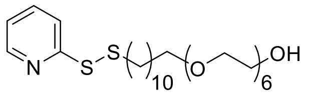

Aldrithiol-2 (1.80 g, 8.17 mmol) and NEt_3 (1.66 mL, 11.9 mmol) were added to a stirred solution of 29-sulfanyl-3,6,9,12,15,18-hexaoxanonacosan-1-ol (2.80 g, 5.97 mmol) in MeOH (50 mL) and the reaction was stirred at rt for ~16 h. The solvent was concentrated *in vacuo* and the crude material was purified by column chromatography (DCM – DCM/MeOH, 95:5) to give the desired product as a colourless oil (2.88 g, 84%). $\mathbf{R_f}$ (DCM:MeOH, 19:1) = 0.56; **IR** (neat, cm^{-1}) 3050 – 2800 (O–H), 1115 (C–O); ^{1}**H NMR** δ (601 MHz, $CDCl_3$) 8.48 – 8.42 (1H, m, Ar*H*), 7.75 – 7.70 (1H, m, Ar*H*), 7.67 – 7.60 (1H, m, Ar*H*), 7.10 – 7.04 (1H, m, Ar*H*), 3.80 – 3.50 (24H, m, 12 × OCH_2CH_2O), 3.44 (2H, t, *J* = 6.8 Hz, $CH_2CH_2CH_2O$), 2.79 (2H, t, *J* = 7.3 Hz, CH_2S), 1.73 (1H, br s, CH_2OH), 1.71 – 1.65 (2H, m, CH_2CH_2), 1.60 – 1.53 (2H, m, CH_2CH_2), 1.40 – 1.22 (14H, m, 7 × $CH_2CH_2CH_2$); ^{13}**C NMR** δ (151 MHz, $CDCl_3$) 160.90 (C), 149.71 (CH), 137.06 (CH), 120.59 (CH), 119.71 (CH), 72.70 (CH_2), 71.69 (CH_2), 70.78 (2 × CH_2), 70.74 (5 × CH_2), 70.71 (CH_2), 70.51 (CH_2), 70.21 (CH_2), 61.91 (CH_2), 39.22 (CH_2), 29.79 (CH_2), 29.70 (CH_2), 29.64 (CH_2), 29.62 (CH_2), 29.60 (CH_2), 29.31 (CH_2), 29.09 (CH_2), 28.64 (CH_2), 26.24 (CH_2); ***m/z*** (ESI+, MeOH) 600 ($[M+Na]^+$, 100%), 578 ($[M+H]^+$, 60).

Step 9: Synthesis of 63-hydroxy-3,6,9,12,15,18,21,46,49,52,55,58,61-tridecaoxa-33,34-dithiatrihexacontanoic acid ("Disulfide SAM OH-COOH")

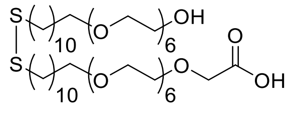

DMAP (0.73 g, 6.00 mmol) and methyl 32-mercapto-3,6,9,12,15,18,21-heptaoxadotriacontanoate (3.25 g, 6.01 mmol) were added to a stirred solution of 1-(pyridin-2-yl)-14,17,20,23,26,29-hexaoxa-1,2-dithiahentriacontan-31-ol (2.88 g, 5.00 mmol) in THF (30 mL) and the reaction was stirred at rt for ~16 h. The solvent was concentrated *in vacuo* and the crude material was purified by column chromatography (DCM – DCM/MeOH, 98:2) to give 63-hydroxy-3,6,9,12,15,18,21,46,49,52,55,58,61-tridecaoxa-33,34-dithiatrihexacontanoic methyl

ester as a colourless solid (2.23 g). The solid was then dissolved in MeOH (30 mL), NaOH (5 mL, 10% Aq) was added dropwise, and the reaction mixture was stirred for 5 h at rt. The reaction mixture was adjusted to ~pH 5 by dropwise addition of acetic acid (1M Aq) and the solvent was concentrated *in vacuo*. The resulting residue was taken up in DCM (100 mL) and washed with brine (2 × 50 mL). The organic phase was dried with $MgSO_4$ and the solvent was removed *in vacuo* to give the desired product as a colourless solid (2.06 g, 42%). **IR** (neat, cm^{-1}) 3100 – 2750 (O–H), 1740 (C=O), 1105 (C–O); ^{1}**H NMR** δ (500 MHz, $CDCl_3$) 4.18 (2H, s, CH_2COOH), 3.76 – 3.58 (48H, m, 24 × OCH_2CH_2O), 3.46 (4H, t, *J* = 6.8 Hz, $CH_2CH_2CH_2O$), 2.75 – 2.63 (4H, m, 2 × CH_2), 1.68 (4H, p, *J* = 7.3 Hz, 2 × CH_2), 1.61 – 1.55 (4H, m, 2 × CH_2), 1.42 – 1.28 (28H, m, 14 × CH_2)(2 × OIH protons not observed); ^{13}**C NMR** δ (126 MHz, $CDCl_3$) 171.50 (C), 72.52 (2 × CH_2), 71.54 (2 × CH_2), 70.63 (2 × CH_2), 70.58 (12 × CH_2), 70.55 (2 × CH_2), 70.35 (2 × CH_2), 70.05 (2 × CH_2), 69.08 (CH_2), 61.74 (2 × CH_2), 39.20 (2 × CH_2), 29.64 (2 × CH_2), 29.58 (2 × CH_2), 29.54 (2 × CH_2), 29.50 (2 × CH_2), 29.49 (2 × CH_2), 29.25 (2 × CH_2), 29.23 (2 × CH_2), 28.54 (2 × CH_2), 26.10 (2 × CH_2); ***m/z*** (ESI+, MeOH) 1016 ($[M+Na]^+$, 100%).

Step 10: Synthesis of 63-hydroxy-N-(2-(prop-2-yn-1-yloxy)ethyl)-3,6,9,12,15,18,21,46,49,52,55,58,61-tridecaoxa-33,34-dithiatrihexacontanamide ("Disulfide SAM OH-propargyl")

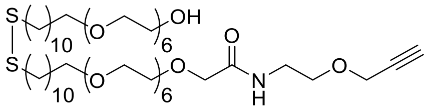

Oyxma (0.22 g, 1.51 mmol) and DIC (0.23 mL, 1.51 mmol) were added to a stirred solution of 63-hydroxy-3,6,9,12,15,18,21,46,49,52,55,58,61-tridecaoxa-33,34-dithiatrihexacontanoic acid (0.75 g, 0.76 mmol) in DCM (5 mL) at rt and the mixture was stirred for 5 mins. A solution of 2-(prop-2-yn-1-yloxy)ethan-1-amine (0.90 g, 0.91 mmol) and DIPEA (0.26 mL, 1.51 mmol) in DCM (2 mL) was then added and the reaction was stirred for a further 2 h. The solvent was concentrated *in vacuo* and the crude material was purified by column chromatography (DCM – DCM/MeOH, 97:3) to give the desired product as a colourless solid (0.32 g, 39%). **IR** (neat, cm^{-1}) 3500 – 3200 (O–H), 1103 (C–O); ^{1}**H NMR** δ (500 MHz, $CDCl_3$) 7.19 (1H, t, *J* = 5.8 Hz, N*H*), 4.13 (2H, d, *J* = 2.4 Hz, OCH_2CCH), 3.97 (2H, s, CH_2CONH), 3.68 – 3.51 (50H, m, 25 × OCH_2), 3.41 (4H, t, *J* = 6.8 Hz, 2 × OCH_2), 2.71 – 2.61 (6H, m, 2 × SCH_2, 1 × NCH_2), 2.45 (1H, t, *J* = 2.4 Hz, CH_2CCH), 1.63 (4H, p, *J* = 7.3 Hz, 2 × CH_2CH_2), 1.57 – 1.50 (4H, m, 2 × CH_2CH_2), 1.35 – 1.22 (28H, m, 14 × CH_2CH_2) (OH proton not observed); ^{13}**C NMR** δ (126 MHz, $CDCl_3$) 170.13 (C), 79.56 (C), 74.87 (CH), 72.63 (CH_2), 71.59 (2 × CH_2), 71.09 (CH_2), 70.68 (4 × CH_2), 70.64 (11 × CH_2), 70.60 (2 ×

CH_2), 70.57 (CH_2), 70.39 (CH_2), 70.32 (CH_2), 70.11 (2 × CH_2), 68.65 (CH_2), 61.77 (CH_2), 58.28 (CH_2), 39.23 (2 × CH_2), 38.58 (CH_2), 29.70 (2 × CH_2), 29.63 (2 × CH_2), 29.59 (2 × CH_2), 29.56 (2 × CH_2), 29.54 (2 × CH_2), 29.30 (2 × CH_2), 29.28 (2 × CH_2), 28.59 (2 × CH_2), 26.15 (2 × CH_2); ***m/z*** (ESI+, MeOH) 1097 ($[M+Na]^+$, 100%).

Step 11: Synthesis of N-(2-(2,5-dioxo-2,5-dihydro-1H-pyrrol-1-yl)ethyl)-63-hydroxy-3,6,9,12,15,18,21,46,49,52,55,58,61-tridecaoxa-33,34-dithiatrihexacontanamide ("Disulfide SAM OH-maleimide")

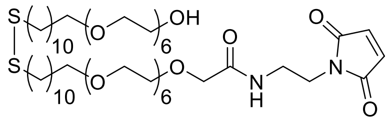

Oyxma (0.29 g, 2.01 mmol) and DIC (0. 13 mL, 2.01 mmol) were added to a stirred solution of 63-hydroxy- 3,6,9,12,15,18,21,46,49,52,55,58,61-tridecaoxa-33,34-dithiatrihexacontanoic acid (1.00 g, 1.01 mmol) in DCM (6 mL) at rt and the mixture was stirred for 5 mins. A solution of 1-(2-aminoethyl)-1H-pyrrole-2,5-dione (0.17 g, 1.21 mmol) and DIPEA (0.35 mL, 2.01 mmol) in DCM (4 mL) was then added and the reaction was stirred for a further 2.5 h. The solvent was concentrated *in vacuo* and the crude material was purified by column chromatography (DCM – DCM/MeOH, 95:5) to give the desired product as a colourless solid (0.55 g, 49%). **IR** (neat, cm^{-1}) 3400 – 3150 (O–H), 1690 (C=O), 1115 (C–O); ^{1}**H NMR** δ (500 MHz, $CDCl_3$) 7.42 (1H, s, N*H*), 6.70 (2H, s, C*H*=C*H*), 3.94 (2H, s, CH_2CONH), 3.89 – 3.52 (50H, m, 25 × OCH_2), 3.49 (2H, q, *J* = 6.0 Hz, CONHCH_2), 3.44 (4H, t, J = 6.8 Hz, 2 × OCH_2), 2.70 – 2.65 (4H, m, 2 × SCH_2), 1.70 – 1.62 (4H, m, 2 × CH_2CH_2), 1.60 – 1.53 (4H, m, 2 × CH_2CH_2), 1.39 – 1.25 (28H, m, 14 × CH_2CH_2) (OH proton not observed); ^{13}**C NMR** δ (126 MHz, $CDCl_3$) 171.18 (C), 170.94 (2 × C), 134.33 (2 × CH), 71.69 (2 × CH_2), 71.04 (CH_2), 70.95 (CH_2), 70.79 (CH_2), 70.74 (2 × CH_2), 70.70 (6 × CH_2), 70.67 (5 × CH_2), 70.61 (2 × CH_2), 70.58 (2 × CH_2), 70.32 (2 × CH_2), 70.16 (2 × CH_2), 68.32 (CH_2), 39.33 (2 × CH_2), 38.02 (CH_2), 37.77 (CH_2), 29.77 (2 × CH_2), 29.72 (2 × CH_2), 29.69 (2 × CH_2), 29.65 (2 × CH_2), 29.63 (2 × CH_2), 29.39 (2 × CH_2), 29.37 (2 × CH_2), 28.68 (2 × CH_2), 26.24 (2 × CH_2); ***m/z*** (ESI+, MeOH) 1138 ($[M+Na]^+$, 100%), 1116 ($[M+H]^+$, 40).

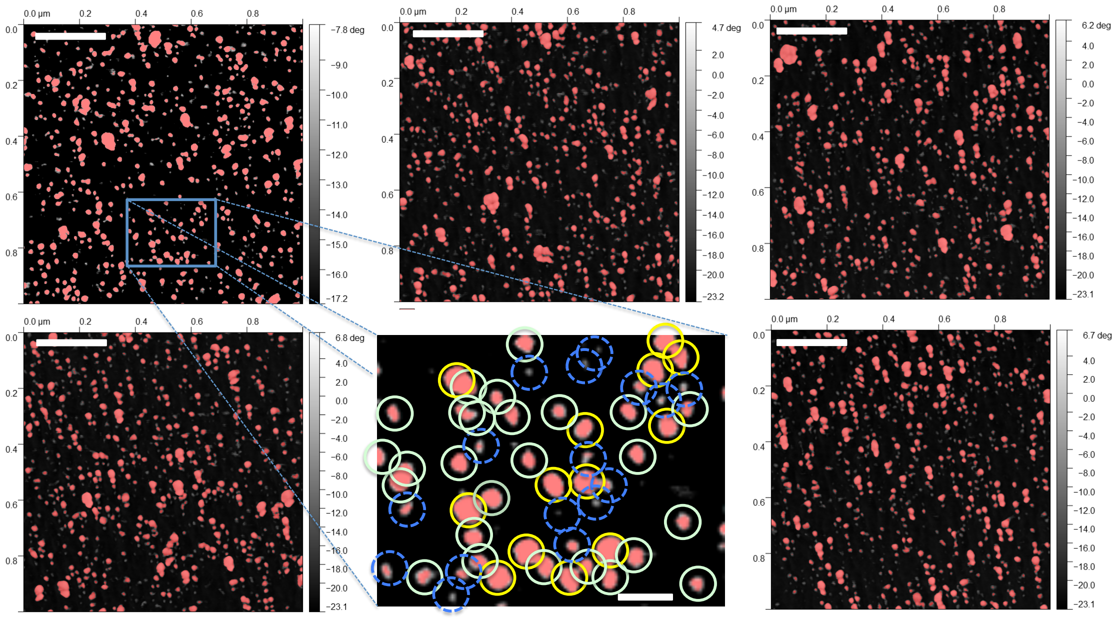

Supplementary Figure 1: *Counting C3b molecules on a C1 sensor chip.* AFM (phase) images of five randomly selected 1-μm-by-1-μm regions within the flow-channel of a C1 chip on which 294 RUs of C3b had been amine coupled in the SPR instrument (scale bar (white) = 0.25 μm). The inset shows an expanded region (scale bar (white) = 0.05 μm). A mask was applied to facilitate automated counting. Automatic counting was performed as described in the text, yielding 490±20 C3b molecules.μm^{-2} (n = 5; hence 490/294 = 1.67 C3b molecules per RU). The blue box and zoomed-in display illustrates a manual count of C4b molecules performed in parallel with automated counting. The 25 pale green circles were assumed to be single C3b molecules while the 13 yellow circles indicate larger features that are likely be instances of two neighbouring C3b molecules not resolved by the AFM tip (summing to 51 C3b molecules in total); blue dotted circles highlight features that are too small for C3b and likely to be artifacts or contaminants. The boxed region measures 0.25 μm by 0.34 μm = 0.085 μm^2, suggesting a density of 1/0.085 x 51 = 600 molecules.μm^{-2}, in reasonable agreement with automated counting.

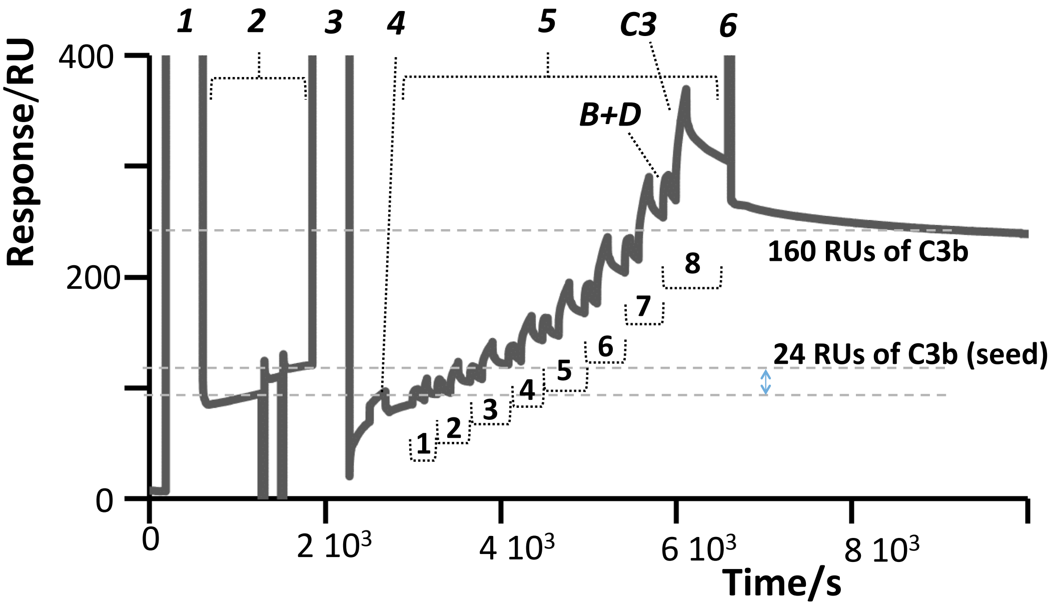

Supplementary Figure 2: *Representative SPR trace for physiological immobilisation of C3b on a C1 chip (chip B)* (also see Fig. 2C). Injections: 1, EDC/NHS (activation); 2, C3b (amine coupling, several injections to achieve 24 RUs); 3, ethanolamine; 4, buffer; 5, eight cycles (numbered) of flowing 50 μM FB mixed with 50 μM FD ("B+D") followed by 50 μM C3 ("3"), thus immobilizing 160 further RUs of C3b; 6, 3 M Nacl.

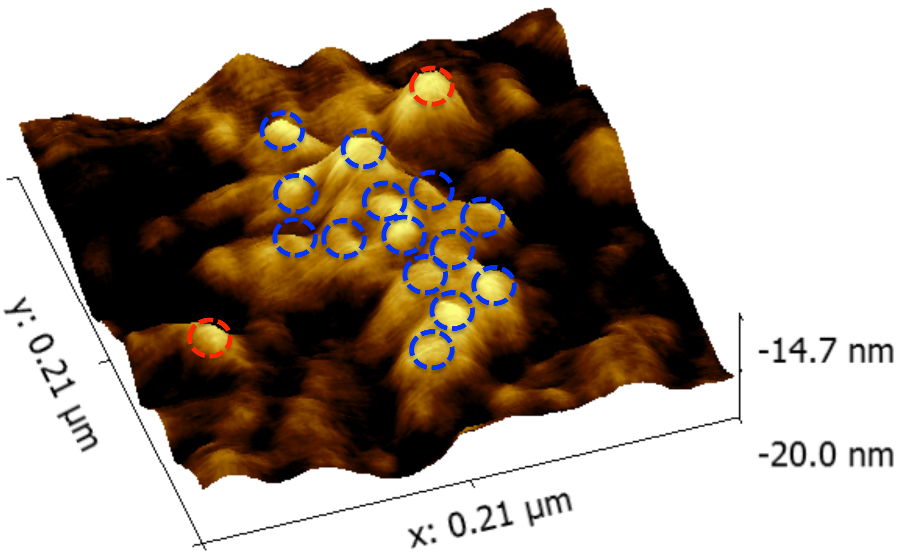

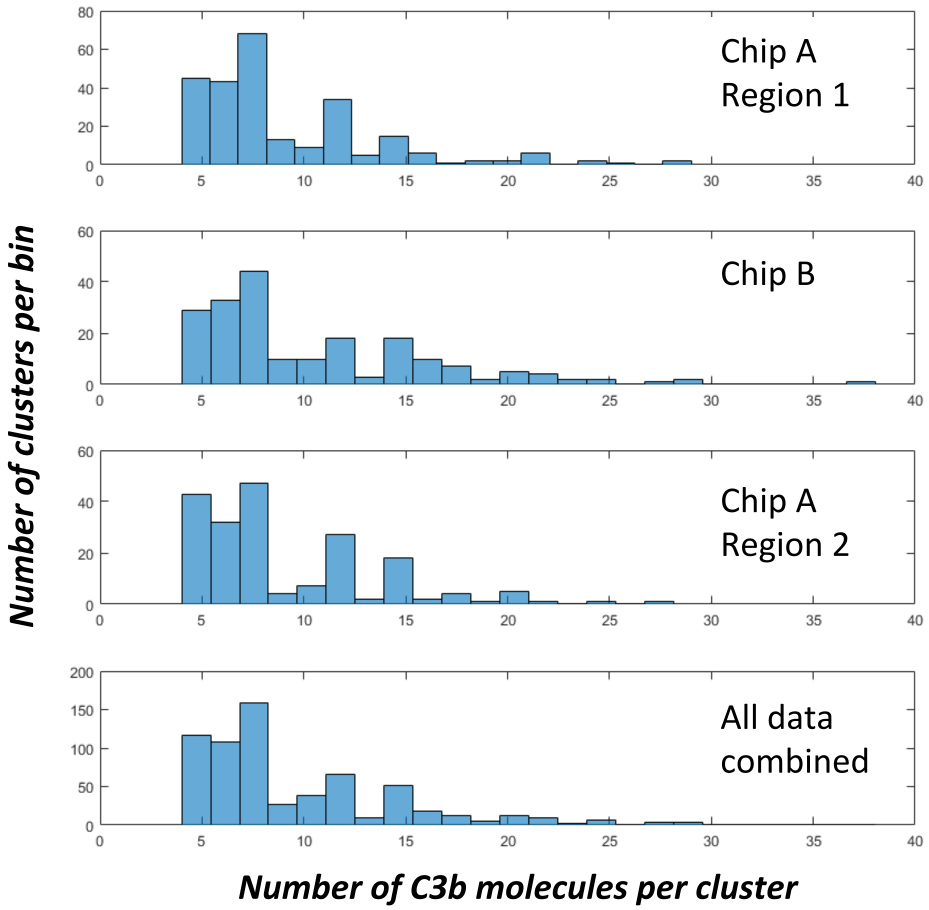

Supplementary Figure 3: *Counting molecules within clusters*. **A.** Combined phase/height image of an example of a cluster of C3b molecules after physiological coupling on a C1 chip. These were counted manually by selecting the taller features. This cluster, measuring ~50 nm x ~150 nm, was estimated to contain 14 molecules (blue circles). The two molecules indicated by red circles were not regarded as part of the cluster. **B.** The numbers of molecules in several hundred clusters were counted on two chips prepared as shown in Figure 2C (chip A, two randomly selected regions) and Supplementary Figure 4 (chip B, one region). In the lower plot all three data sets are combined.

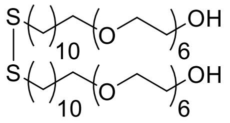

Disulfide SAM OH-OH

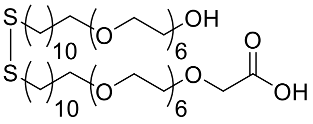

Disulfide SAM OH-COOH

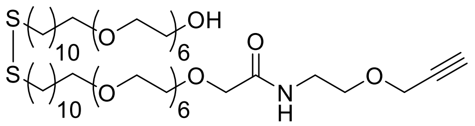

Disulfide SAM OH-Propargyl

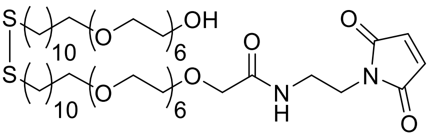

Disulfide SAM OH-Maleimide

Supplementary Figure 4 *Chemical structures of SAM components*. These were synthesized according to the procedures in the previous section of Supplementary Information. Each disulfide-linked undecanethiol SAM component is named by the chemical natures of its two head groups *e.g.* OH-COOH has a hydroxyl and a carboxyl head group.

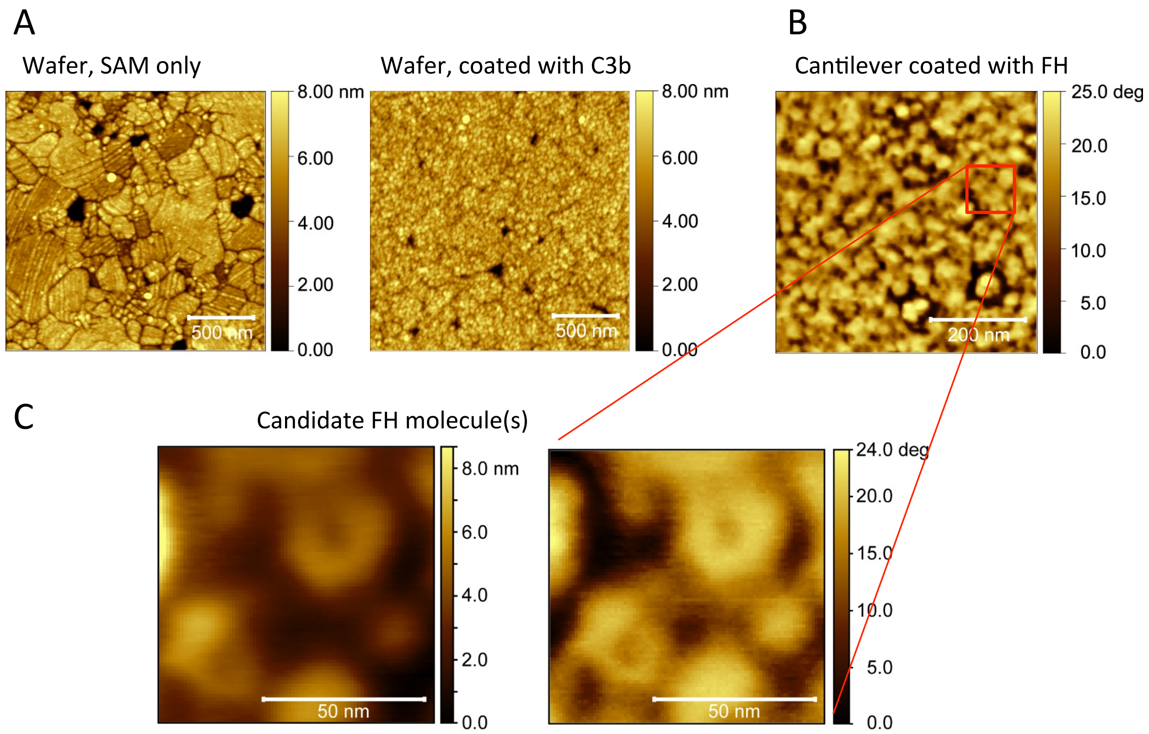

Supplementary Figure 5. *AFM-derived images of wafer and cantilever (tip) surfaces used in force-distance measurements.* **A.** AFM (topography) images of the SAM-coated gold wafer before (left) and after attachment using amine coupling of C3b (right) (see Methods). The images are consistent with the desired dense covering of C3b molecules. **B.** AFM (phase) image of the cantilever. The cantilever was dipped in a series of solutions that were expected to leave FH molecules anchored to amine-coupled PspCN (see Methods) on its SAM-coated surface. **C.** Zooming in on a typical object on the FH-decorated cantilever as seen in B. The lateral resolution in AFM is limited by the sharpness of the tip (the convolution effect) as well as the nature of the object to be imaged, making objects appear bulkier than they are in reality. These images are consistent with a high density of FH molecules on the cantilever and hence on the tip.

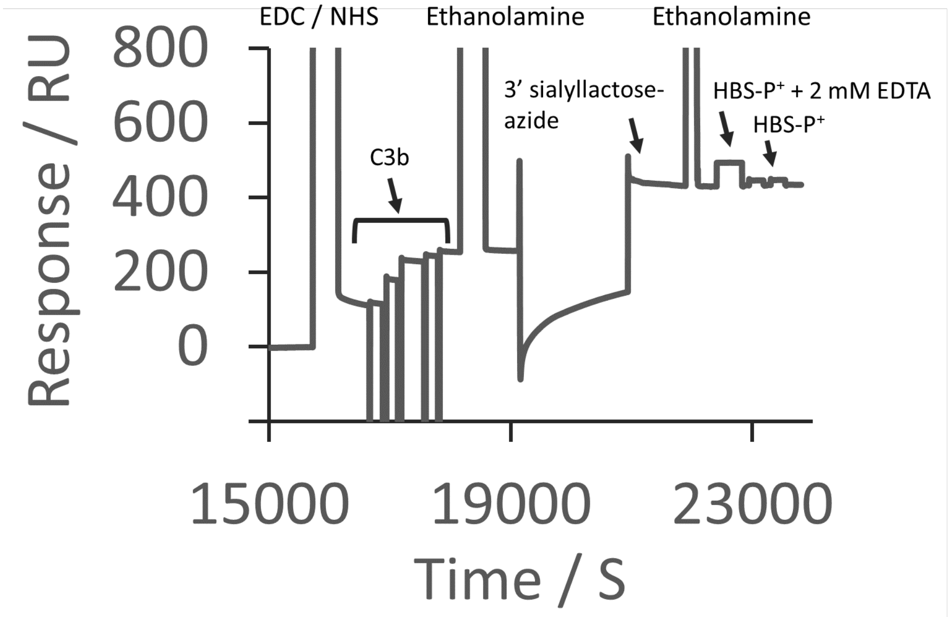

Supplementary Figure 6: *Preparation of the 3'-sialyllactose- and C3b-decorated HJEM 7 surface*. The surface of the HJEM7 sensorchip consisted of a SAM with both carboxyl and propargyl head groups (Table I). In the SPR instrument, C3b was flowed over the HJEM7 chip, following activation of carboxyl groups with EDC/NHS. This process was repeated until 150 RUs of C3b had been amine coupled on the chip. After an injection of ethanolamine to cap any remaining activated esters, 3'-sialylactose-azide was flowed over the chip and attached *via* the propargyl groups on the chip in a copper-catalysed azide-alkyne 1,3-dipolar cycloaddition. The chip was then washed with buffer and EDTA.
